# Modulating the Band Structure of Metal Coordinated Salen COFs and an In Situ Constructed Charge Transfer Heterostructure for Electrocatalysis Hydrogen Evolution

**DOI:** 10.1002/advs.202105912

**Published:** 2022-06-03

**Authors:** Boying Zhang, Liling Chen, Zhenni Zhang, Qing Li, Phathutshedzo Khangale, Diane Hildebrandt, Xinying Liu, Qingliang Feng, Shanlin Qiao

**Affiliations:** ^1^ College of Chemistry and Pharmaceutical Engineering Hebei University of Science and Technology Shijiazhuang 050018 P. R. China; ^2^ Department of Chemical Engineering Faculty of Engineering and the Built Environment University of Johannesburg Doornfontein 2028 South Africa; ^3^ Key Laboratory of Special Functional and Smart Polymer Materials of Ministry of Industry and Information Technology School of Chemistry and Chemical Engineering Northwestern Polytechnical University Xi'an 710072 P. R. China; ^4^ African Energy Leadership Centre WITS Business School and Molecular Science Institute School of Chemistry University of Witwatersrand Johannesburg 2050 South Africa; ^5^ Institute for Development of Energy for African Sustainability University of South Africa Florida 1709 South Africa; ^6^ Hebei Electronic Organic Chemicals Technology Innovation Center Shijiazhuang 050018 P. R. China

**Keywords:** band structure modulation, heterostructures, hydrogen evolution reaction, in situ solid‐state polymerization, Salen covalent organic frameworks (COFs)

## Abstract

A series of crystalline, stable Metal (Metal = Zn, Cu, Ni, Co, Fe, and Mn)‐Salen covalent organic framework (COF)_EDA_ complex are prepared to continuously tune the band structure of Metal‐Salen COF_EDA_, with the purpose of optimizing the free energy intermediate species during the hydrogen evolution reaction (HER) process. The conductive macromolecular poly(3,4‐ethylenedioxythiophene) (PEDOT) is subsequently integrated into the one‐dimensional (1D) channel arrays of Metal‐Salen COF_EDA_ to form heterostructure PEDOT@Metal‐Salen COF_EDA_ via the in situ solid‐state polymerization method. Among the Metal‐Salen COF_EDA_ and PEDOT@Metal‐Salen COF_EDA_ complexes, the optimized PEDOT@Mn‐Salen COF_EDA_ displays prominent electrochemical activity with an overpotential of 150 mV and a Tafel slope of 43 mV dec^−1^. The experimental results and density of states data show that the continuous energy band structure modulation in Metal‐Salen COF_EDA_ has the ability to make the metal *d*‐orbital interact better with the *s*‐orbital of H, which is conducive to electron transport in the HER process. Moreover, the calculated charge density difference indicates that the heterostructures composed of PEDOT and Metal‐Salen COF_EDA_ induce an intramolecular charge transfer and construct highly active interfacial sites.

## Graphical abstract

The conductive macromolecule cables are subsequently integrated into the one‐dimensional (1D) channels of a series of crystalline Metal‐Salen covalent organic frameworks (COFs) (Metal = Zn, Cu, Ni, Co, Fe, and Mn) to form heterogenous poly(3,4‐ethylenedioxythiophene)@Metal‐Salen COF_EDA_ complexes via the in situ solid‐state polymerization method, in order to improve the electrochemical activity of hydrogen evolution reaction.

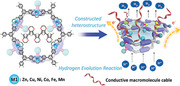

## 1 Introduction

The increasing depletion of energy and the deterioration of the natural environment are driving people to look for environmentally friendly and sustainable energy sources to replace the traditional nonrenewable resources in use.^[^ ^1^, ^2^, ^3^ ^]^ Hydrogen energy has the characteristics of light weight, satisfactory thermal conductivity, zero carbon dioxide emissions, and a high calorific value, which provides an alternative energy for clean development.^[^ ^4^, ^5^, ^6^ ^]^ The hydrogen evolution reaction (HER) is a method that is being researched to obtain high purity hydrogen. It has become a popular topic of research in the energy conversion technology field, and has attracted increasing attention in recent years.^[^ ^7^, ^8^, ^9^ ^]^ Platinum provides the optimum Gibbs free energy (Δ*G*_H*_) and quick reaction dynamics and is generally acknowledged as being the most effective electrocatalyst for HER.^[^ ^10^ ^]^ However, the scarcity of this precious metal and its high cost hinder progress being made with it being used as a catalyst for HER, which has led to research into the development of more cost‐efficient non‐noble metal electrocatalysts.^[^ ^11^ ^]^

The HER process takes place on the surface of the electrode, while mass transport in the electrocatalyst comes about through apertures.^[^ ^12^ ^]^ Electrode material with rich porosity and a large specific surface area plays a crucial role in HER. Recently, porous carbon materials that are generally used as a support have been extensively researched in terms of HER because of their excellent conductivity and abundant porosity, which ensures the required electron transfer and adequate active sites.^[^ ^13^, ^14^, ^15^, ^16^, ^17^ ^]^ However, porous carbon materials are usually prepared at a high temperature, which requires extensive energy consumption and the chemical composition and structure becomes unpredictable and uncontrollable.^[^ ^18^ ^]^

Compared with porous carbon, covalent organic frameworks (COFs) are a kind of porous organic polymer, which is assembled by connecting organic building blocks through dynamic covalent bonds. They possess a crystalline structure and an aligned one‐dimensional (1D) channel.^[^ ^19^ ^]^ On account of the modular nature of COFs, the pore structure and functional groups can be precisely regulated according to reticular chemistry.^[^ ^20^ ^]^ This means that well‐defined active sites can be accurately introduced into long‐range ordered channels and the accessible surface of COFs.^[^ ^21^ ^]^ Therefore, COFs can serve as a potential electrocatalyst model for exploring the design of electrocatalysts at the molecular level and even the atomic level.^[^ ^22^ ^]^

Recently, various COF‐based electrocatalysts have been proposed, which facilitate fundamental research and practical applications in terms of HER. As the first example of a COF‐based electrocatalyst for HER, a 2D SB‐PORPy COF with porphyrin and pyrene units was prepared as a metal‐free electrocatalyst for HER, in order to explore the hydrogen production process using imine nitrogen as the active site.^[^ ^23^ ^]^ The intrinsic SB‐PORPy COF shows a hysteretic onset potential of 50 mV and poor cyclic stability within 500 cycles. To further enhance the electrochemical performance of HER, Xia and co‐workers developed a series of electrocatalysts by annealing a complex of COF compound coordinated with Ru. Ru@C_4_N displays excellent electrochemical activity for HER, and surpasses that of the commercial Pt/C electrocatalyst.^[^ ^24^ ^]^ In our previous work, we reported a series of electrocatalysts adopting intrinsic covalent triazine frameworks as the carriers to support metal ions for HER.^[^ ^25^ ^]^ Both the experimental results and the theoretical calculations revealed that the synergetic effect of the intrinsic porosity and coordinate metals–N improved the electrocatalytic kinetics and stability of HER. Despite the progress made with COF‐based electrocatalysts, most of the COF‐based electrocatalysts that show excellent performance have undergone high temperature treatment during preparation, which is not conducive to understanding the structure–activity relationship.

Recently, porphyrin and phthalocyanine‐based COFs have exhibited excellent electrocatalytic activity for electrocatalysts because of their regulable coordination environment and clearly‐defined active sites.^[^ ^26^, ^27^ ^]^ Similarly, *N*,*N*′‐bis(salicylidene)ethylenediamine (Salen) with the ability to stabilize metal ions has a coordination environment similar to porphyrin and phthalocyanine. Moreover, Salen units are not only ligands in coordination chemistry, but also “locators” in reticular chemistry. When Salen ligands are coordinated with metal ions during one‐pot solvothermal synthesis, macrocyclic compounds with a rigid structure can be formed, which can enhance the rigidity of the linkers and reduce their spatial freedom, which is conducive to the formation of single crystals. Salen‐based COFs have displayed high crystallinity and well‐defined single metal atomic sites that favor increasing the utilization of active sites for catalytic processes.^[^ ^28^ ^]^ Therefore, Salen COFs can serve as ideal models for studying the structure–activity relationship of electrocatalysts.

**Scheme 1:**
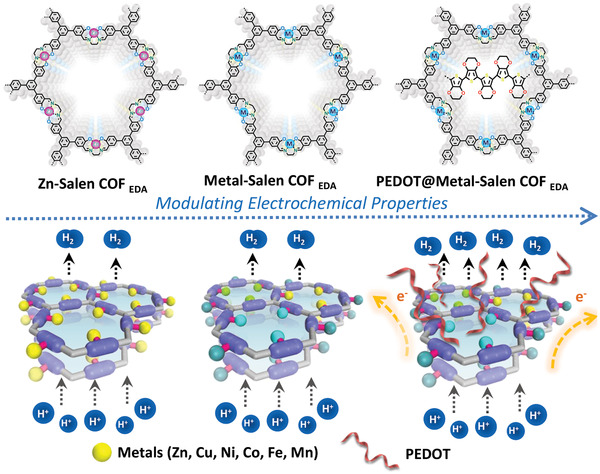
Schematics of as‐synthesized Metal‐Salen COF_EDA_ and PEDOT@Metal‐Salen COF_EDA_ as electrocatalysts for HER.

In this paper, a crystalline novel Zn‐Salen COF_EDA_ was prepared by means of the Schiff base condensation reaction between 1,3,5‐tris(4′‐hydroxy‐5′‐formylphenyl)benzene (THB) and ethylenediamine. For the purpose of band structure modulation, Zn^2+^ is exchanged with other metal ions to obtain a series of Metal‐Salen COF_EDA_ (Metal = Cu, Ni, Co, Mn, and Fe). Conductive macromolecule poly(3,4‐ethylenedioxythiophene) (PEDOT) has outstanding electrical characteristics and chemical stability. It is integrated into the 1D channels of Metal‐Salen COFs as an electronic transmission cable to improve the conductivity of composites by means of the in situ solid‐state polymerization method detailed in previous reports.^[^ ^29^ ^]^ Finally, the Metal‐Salen COF_EDA_ and PEDOT@Metal‐Salen COF_EDA_ obtained as electrocatalysts of HER were evaluated. A schematic of this process is shown in **Scheme** **1**. The PEDOT@Mn‐Salen COF_EDA_ heterostructure displays prominent electrochemical activity with an overpotential of 150 mV and a Tafel slope of 43 mV dec^−1^. The PEDOT@Metal‐Salen COF_EDA_ is markedly enhanced compared to Metal‐Salen COF_EDA_. The experimental results, density of states (DOS), Δ*G*_H*_, and charge density difference data reveal the relationship between HER activity and band structure. The continuous band structure modulation in Metal‐Salen COF_EDA_ enables the metal *d*‐orbital to interact better with the *s*‐orbital of H. Moreover, the heterostructure of PEDOT@Metal‐Salen COF_EDA_ induces the intramolecular charge transfer and the construction of interfacial active sites with high activity, which contribute to the HER process.

## 2 Results and Discussion

**Figure 1:**
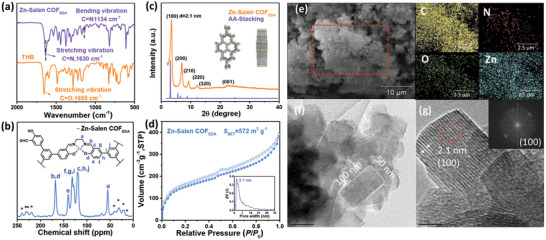
a) FT‐IR spectrum of Zn‐Salen COF_EDA_. b) Solid‐state ^13^C CP‐MAS NMR spectrum of Zn‐Salen COF_EDA_. (The asterisks indicate the side‐peaks.) c) P‐XRD pattern of Zn‐Salen COF_EDA_. The insets are the top and side views of Zn‐Salen COF_EDA_ that were simulated using Materials Studio. d) N_2_ adsorption/desorption isotherms. The inset is the pore width distribution plot of Zn‐Salen COF_EDA_. e) SEM images and EDS element mapping images of Zn‐Salen COF_EDA_. f,g) HR‐TEM image of Zn‐Salen COF_EDA_. The inset in (g) displays the fast Fourier transform of the image.

A crystalline Zn‐Salen COF_EDA_ complex was synthesized by co‐polycondensation of THB and ethane diamine with the assistance of Zn(OAC)_2_·2H_2_O in the solvent mixture comprising a mesitylene, EtOH, and acetic acid aqueous solution (6 mol L^−1^) via solvothermal method at 120 °C for 3 days. The yellow crystalline powder was obtained in ≈95% yield. It is worth noting that the pivotal building block is THB, which can be synthesized using commercially available compounds. (Please see the Supporting Information for details.) The Fourier transform infrared spectroscopy (FT‐IR) spectrum of Zn‐Salen COF_EDA_ (**Figure** **1**a) shows that the characteristic C═O stretching vibration band disappears at 1655 cm^−1^ and new generation of C═N linkages appear at 1630 and 1134 cm^−1^. This demonstrates successful polycondensation between THB and ethylenediamine. The solid‐state ^13^C cross‐polarization/magic‐angle spinning nuclear magnetic resonance (CP/MAS NMR) signals of Zn‐Salen COF_EDA_ can be definitely assigned as the proposed structure (Figure 1b). Obviously, the typical signal is obtained from the peak at 168 ppm, which corresponds to C═N and is indicative of the formation of the imine bond. This result is consistent with that of FT‐IR. In addition, the high resolution and narrow half‐peak width of these peaks in the solid‐state ^13^C CP/MAS NMR spectrum indicate that the chemical environment of Zn‐Salen COF_EDA_ is homogeneous, which reflects the well‐organized structure.^[^ ^28^ ^]^

The crystalline structure of Zn‐Salen COF_EDA_ is resolved by means of powder X‐ray diffraction (P‐XRD). The computational structural simulation was carried out using Materials Studio. It was observed that the simulated P‐XRD pattern of the eclipsed stacking (AA stacking) with cell parameters of *a* = *b* = 28.81 Å, *c* = 3.62 Å, *α* = *β* = 90.00°, *γ* = 120.00° offers a good fit for the experimentally obtained diffraction patterns shown in Figure 1c. The Zn‐Salen COF_EDA_ displays an intense diffraction peak at 3.43°, 6.99°, 9.27°, 12.18°, 15.18°, and 22.68°, which are assigned to the (100), (200), (210), (220), (320), and (001) facets.

The high‐resolution transmission electron microscope (HR‐TEM) images are shown in Figure 1f,g. Figure 1f shows the stacked lamellar morphology and single crystals with a size of 100 × 50 nm of Zn‐Salen COF_EDA_. Figure 1g shows the lattice fringe of 2.1 nm that corresponds to the (100) facets of the hexagonal lattice. This is consistent with the P‐XRD and simulation results of Zn‐Salen COF_EDA_. These results show that Zn‐Salen COF_EDA_ has a high crystal quality and a well‐organized AA stacked structure. The porosity of the Zn‐Salen COF_EDA_ was measured by N_2_ adsorption/desorption isotherms at 77 K, as shown in Figure 1d. The Zn‐Salen COF_EDA_ isotherm is a type‐IV isotherm with the characteristics of mesoporous materials, as indicated by International Union of Pure and Applied Chemistry. The Brunauer–Emmett–Teller (BET) surface area of Zn‐Salen COF_EDA_ is calculated to be 572 m^2^ g^−1^. The pore size distribution of Zn‐Salen COF_EDA_ was calculated by means of quenched solid density functional theory (QS‐DFT). It was centered at 2.1 nm, which corresponds to the lattice fringe of (100) facets and the simulated pore width. The morphology features and element distribution of Zn‐Salen COF_EDA_ were observed by scanning electron microscope (SEM) and energy‐dispersive X‐ray spectra (EDS). Zn‐Salen COF_EDA_ shows a cotton‐like morphology and homogeneous distribution of elements (see Figure 1e). When zooming in further, it can be seen that the morphology is composed of Zn‐Salen COF_EDA_ rods with a size of about 1000 × 100 nm (Figure S3, Supporting Information).

**Figure 2:**
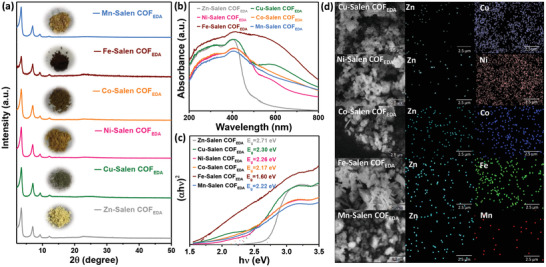
a) P‐XRD patterns and photographs of Zn‐Salen COF_EDA_ after postsynthetic metal ion exchange. b) UV–vis DRS spectrum for Metal‐Salen COF_EDA_ measured in the solid state. c) The Tauc plots and corresponding bandgap of Metal‐Salen COF_EDA_. d) SEM images and EDS element mapping images of Metal‐Salen COF_EDA_.

The readily accessible 1D channel array in Zn‐Salen COF_EDA_ and the dynamic Zn—O_2_—N_2_ bonds allow for the possibility of metal ion exchange. Therefore, the as‐synthesized Zn‐Salen COF_EDA_ was impregnated in a saturated solution of metallic salts, including Cu(OAC)_2_·H_2_O, Ni(OAC)_2_·4H_2_O, Co(OAC)_2_·2H_2_O, Fe(OAC)_2_·H_2_O, and Mn(OAC)_2_·4H_2_O for 2 days, after which Metal‐Salen COF_EDA_ complexes were obtained (see the Supporting Information for details). As seen in **Figure** **2**a, P‐XRD revealed that the Metal‐Salen COF_EDA_ retained the original crystal structure and high crystallinity after ion exchange. It is also evident that the appearance of the Metal‐Salen COF_EDA_ varies from light yellow to dark, which indicates the feasibility of ion exchange.

The Metal‐Salen COF_EDA_ were explored optical/electrical characteristic using ultraviolet–visible diffuse reflectance (UV–vis DRS) spectroscopy. The UV–vis spectra in Figure 2b show that the Zn‐Salen COF_EDA_ exhibits a characteristic absorption band at 470 nm, while other Metal‐Salen COF_EDA_ displays a significant red shift with long‐tail absorption band that extends to 800 nm. The wide range of absorption is explained by the rapid in‐plane electron migration caused by hybridization of the electron cloud in the polymer framework and the extended *π*‐electron delocalization from the electron‐donating Salen ligand to the electron‐withdrawing metal unoccupied orbital.^[^ ^30^ ^]^ Using the Tauc plots, the bandgaps of Zn‐Salen COF_EDA_, Cu‐Salen COF_EDA_, Ni‐Salen COF_EDA_, Co‐Salen COF_EDA_, Fe‐Salen COF_EDA_, and Mn‐Salen COF_EDA_ were estimated to be 2.71, 2.30, 2.26, 2.17, 1.60, and 2.22 eV, respectively. It follows that metal ion exchange enables regulation of the bandgaps of Metal‐Salen COF_EDA_. This is explained by i) the electron‐donating Salen ligand and the electron‐withdrawing metal unoccupied orbital can easily construct a donor–acceptor (D–A) type semiconductor. ii) The generated electronic push–pull effect is beneficial for narrowing the bandgap of the polymers.^[^ ^31^, ^32^ ^]^ In order to understand the degree of ion exchange, element distribution is determined by means of SEM–EDS. In Figure 2d and Figure S4 (Supporting Information), the homogeneous element distribution is clearly visible. Notably, residual Zn^2+^ in other Metal‐Salen COF_EDA_ is not completely exchanged, which is consistent with previous reports.^[^ ^33^ ^]^

**Figure 3:**
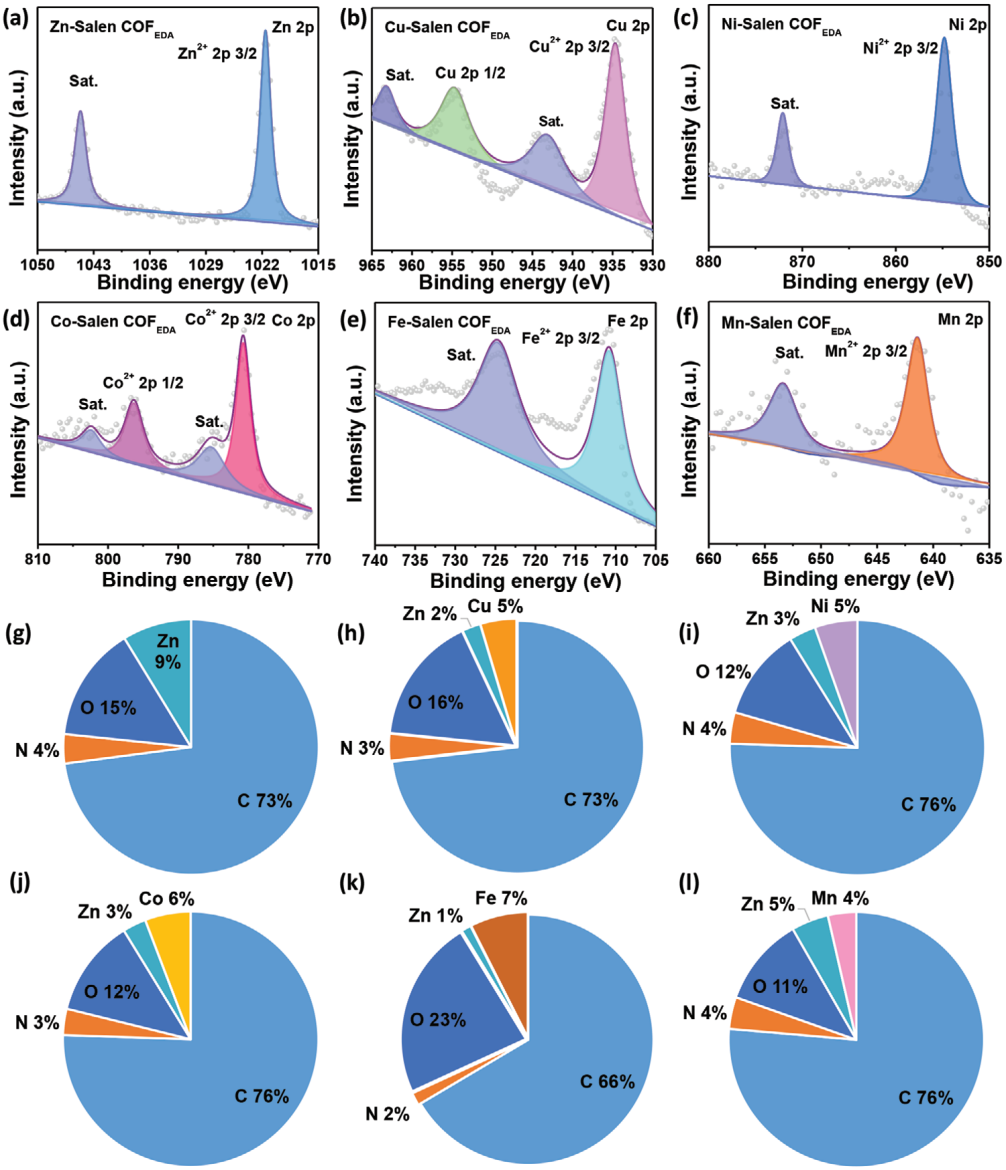
a–f) XPS spectra of Metal‐Salen COF_EDA_. g–l) Surface concentration of elements obtained by XPS.

The surface concentration and chemical state of the elements are determined by X‐ray photoelectron spectroscopy (XPS). The XPS spectra (Figure S5, Supporting Information) reveal that C, N, O, and the corresponding metal ions consist of Metal‐Salen COF_EDA_, while a certain amount of Zn^2+^ remains in the framework after ion exchange (**Figure** **3**g–l). This is related to the kinetic equilibrium of the coordination bonds.^[^ ^33^ ^]^ It is further indicated that the Cu, Ni, Co, Fe, and Mn atoms are successfully introduced into Zn‐Salen COF_EDA_. The N 1s, O 1s, and Zn 2p high‐resolution XPS spectra of Metal‐Salen COF_EDA_ are shown in Figure S6 of the Supporting Information. The deconvoluted N 1s spectra of Metal‐Salen COF_EDA_ show two peaks—at 398.5 ± 0.2 and 399.5 ± 0.3 eV—which correspond to M—N and C═N, respectively.^[^ ^34^ ^]^ The O 1s spectra of Metal‐Salen COF_EDA_ were deconvoluted into two peaks at 530.8 ± 0.7 and 532.0 ± 0.5 eV, which correspond to O—M and C—O.^[^ ^34^ ^]^ In the deconvoluted metals 2p spectra of Metal‐Salen COF_EDA_, two sets of signals are observed, which correspond to M^2+^ 2p_3/2_ and the satellite signal. This indicates that the metal species are maintained in a +2 state (Figure 3a–f).^[^ ^33^, ^35^ ^]^ Furthermore, compared to the metallic precursors, the binding energy of the metal ions shifts upward, which indicates electron transfer from the metals to the COF_EDA_.^[^ ^36^ ^]^ The change in the binding energy indicates that there is strong interaction between the metal and the COF_EDA_ skeleton, which stabilizes single metal ions and also leads to redistribution of the charge through coordinated electron transfer.^[^ ^25^, ^37^ ^]^ Together, these XPS data further confirmed that exchanged Salen COF_EDA_ were successfully synthesized.

**Figure 4:**
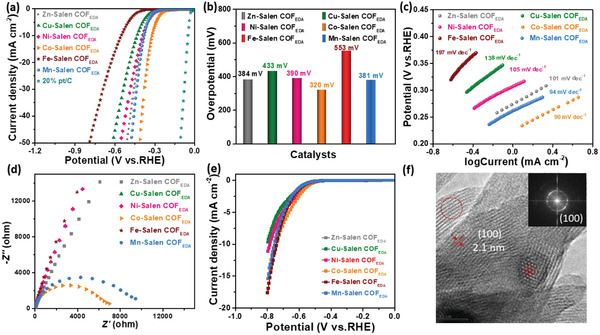
Electrochemical performance of Metal‐Salen COF_EDA_. a) LSVs of the catalysts in a H_2_SO_4_ aqueous solution (0.5 mol L^−1^). b) Overpotential of the electrocatalysts in this work. c) Tafel slopes of the Metal‐Salen COF_EDA_ electrocatalysts calculated from the LSVs. d) EIS spectra of Metal‐Salen COF_EDA_. e) LSVs of the Metal‐Salen COF_EDA_ in a H_2_SO_4_ aqueous solution with SCN^−^ (0.5 mol L^−1^). f) TEM image of Co‐Salen COF_EDA_ after the stability test. (The inset displays the fast Fourier transform (FFT) of the image.)

The electrocatalytic HER performance of Metal‐Salen COF_EDA_ was evaluated by linear sweep voltammetry (LSV) using a typical three‐electrode system in a H_2_SO_4_ aqueous solution (0.5 mol L^−1^, N_2_ saturated). In addition, commercial 20% Pt/C was used as a reference electrocatalyst. As shown in **Figure** **4**a, the LSV curves of Metal‐Salen COF_EDA_ reveal that the cathode current increases dramatically at a certain potential, which indicates that the electrocatalysts are activated on the electrode surface. Co‐Salen COF_EDA_ shows the best electrocatalytic HER activity, with an overpotential of 320 mV at 10 mA cm^−2^, compared to Zn, Cu, Ni, Fe, and Mn‐Salen COF_EDA_ (Figure 4b). This may be explained by the fact that the electron‐withdrawing metal unoccupied orbit gives the Co center greater reducing power, which shifts the water reduction potential positively and lowers the overpotential of HER.^[^ ^38^ ^]^

In order to investigate the electrocatalytic mechanism and kinetics of Metal‐Salen COF_EDA_, the Tafel slope was obtained from the LSV curves. The Tafel plots shown in Figure 4c show the lowest slope for Co‐Salen COF_EDA_ (90 mV dec^−1^), followed by Mn‐Salen COF_EDA_ (94 mV dec^−1^), Zn‐Salen COF_EDA_ (101 mV dec^−1^), Ni‐Salen COF_EDA_ (105 mV dec^−1^), Cu‐Salen COF_EDA_ (138 mV dec^−1^), and Fe‐Salen COF_EDA_ (197 mV dec^−1^). This indicates that Co‐Salen COF_EDA_ has fast electrocatalytic kinetics. Electrochemical impedance spectroscopy (EIS) spectra were obtained to characterize the charge transfer at the interface electrode/electrolyte. The Co‐Salen COF_EDA_ shows the least resistance charge transfer (*R*_ct_) among the series of electrocatalysts in this work (Figure 4d), which indicates that Co‐Salen COF_EDA_ has the fastest charge transfer at the interface between the electrode and the electrolyte.^[^ ^39^ ^]^

Cyclic voltammetry (CV) and chronoamperometry (*V*–*t*) were used to measure the stability of Co‐Salen COF_EDA_, as shown in Figure S7 of the Supporting Information. As shown in Figure S7a of the Supporting Information, the last polarization curve almost overlaps the first cycle after 3000 cycles. In Figure S7b of the Supporting Information, Co‐Salen COF_EDA_ exhibited a very steady potentiometric curve for 72 h of continuous operation, which indicates the durable stability of Co‐Salen COF_EDA_. Moreover, after stability measurements, the TEM images of Co‐Salen COF_EDA_ show clear lattice fringes, which indicate that Co‐Salen COF_EDA_ retained the pristine crystal structure and high crystallinity (Figure 4f; Figure S8, Supporting Information). The changes in the surface electronic states of Co‐Salen COF_EDA_ after HER were investigated by means of XPS analysis (Figure S9, Supporting Information). In high‐resolution Co 2p spectrum, Co species are remained in a +2 state, meanwhile, no new species emerged from the Co‐Salen COF_EDA_ after HER, which indicates that active species remain unchanged. These electrochemical testing methods and physical characterization together proved the stability of the Co‐Salen COF_EDA_. In order to verify the active site in Metal‐Salen COF_EDA_ and determine the contribution of metal ions toward the HER, the thiocyanate ions (SCN^−^) poisoning experiments of Metal‐Salen COF_EDA_ were carried out in an acidic electrolyte. As shown in Figure 4e, the cathode current of the Metal‐Salen COF_EDA_ decreased dramatically after the addition of SCN^−^. This indicates that the activity of HER mainly comes from the contribution of metal ions. The improvement in the performance of HER is attributed to adjust interaction between the active site and COF_EDA_ by virtue of different metal ions.

According to the above analysis, the aligned channels of Metal‐Salen COF_EDA_ allow unimpeded transfer of guest molecules, which increases the accessibility of the active site. Also, Salen complexes as active centers are capable of regulating the coordination environment and local structure flexibly with the help of metal ion exchange.^[^ ^33^, ^40^ ^]^ Therefore, these unique characteristics enable Metal‐Salen COF_EDA_ as a model to be researched structure–activity relationship in electrocatalysis (see Section 3 for an explanation). However, the electrocatalytic performance of Metal‐Salen COF_EDA_ may be limited by conductivity. Because the polarized imine bond and the flexible unconjugated ethyl group in the diamine linking‐group limit electron delocalization.

Some research has been done on conductive COFs being synthesized using a bottom‐up strategy, but these conductive COFs are strictly limited by the species of building blocks.^[^ ^41^, ^42^, ^43^ ^]^ Additionally, some conductive carriers—such as carbon nanotubes and reduced graphene oxide—have been introduced in COFs to enhance conductivity.^[^ ^44^, ^45^ ^]^ However, these conductive carriers mostly exist on the outer surface of COFs through weak van der Waals forces. Therefore, we integrated the PEDOT conductive 1D linear polymer into 1D channel arrays of Metal‐Salen COF_EDA_ using the in situ solid state polymerization method, in order to improve electron transfer efficiency, and so promote HER performance.

**Figure 5:**
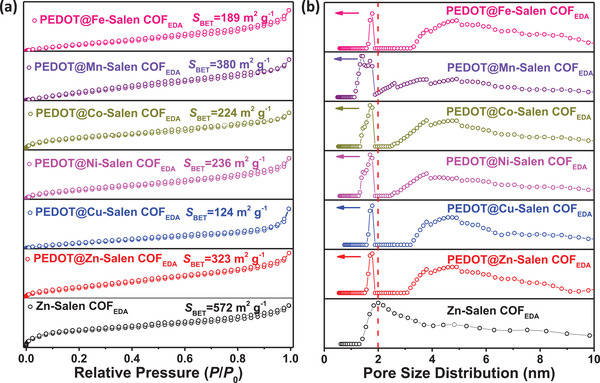
a) N_2_ adsorption/desorption isotherms and b) pore width distribution plots of PEDOT@Metal‐Salen COF_EDA_.

The in situ solid state polymerization method was used to construct heterostructure composites, which are labeled PEDOT@Metal‐Salen COF_EDA_. (The detailed synthesis steps are shown in the Supporting Information). The FT‐IR spectra of the PEDOT@Metal‐Salen COFE_DA_ (Figure S10a, Supporting Information) show the asymmetric C═C stretching vibration band and inter‐ring C—C stretching vibration band at 1475 and 1315 cm^−1^. This demonstrates successful polycondensation of PEDOT.^[^ ^29^ ^]^ In addition, the imine bond (1630 cm^−1^) is still clearly visible in the PEDOT@Metal‐Salen COF_EDA_, which is a preliminary indication that PEDOT has been successfully polymerized into Metal‐Salen COF_EDA_. The crystalline structure of PEDOT@Metal‐Salen COF_EDA_ was determined by means of P‐XRD. As shown in Figure S10b of the Supporting Information, compared with the Metal‐Salen COF_EDA_, the intensity of the diffraction peak that corresponds to the (100) crystal plane is reduced in PEDOT@Metal‐Salen COF_EDA_. This can be explained by the presence of amorphous linear PEDOT in the pores of the Metal‐Salen COF_EDA_. The BET surface area was calculated from the N_2_ adsorption/desorption isotherms of PEDOT@Metal‐Salen COF_EDA_. The BET surface area of PEDOT@Zn‐Salen COF_EDA_, PEDOT@Cu‐Salen COF_EDA_, PEDOT@Ni‐Salen COF_EDA_, PEDOT@Co‐Salen COF_EDA_, PEDOT@Fe‐Salen COF_EDA_, and PEDOT@Mn‐Salen COF_EDA_ are 323, 124, 236, 224, 380, and 189 m^2^ g^−1^, respectively (**Figure** **5**a).

The SEM–EDS images (Figure S11, Supporting Information) show the uniform distribution of the S and Br elements in PEDOT@Metal‐Salen COF_EDA_. This provides evidence of successful construction of the heterojunction in the nanospace channels. More importantly, as the appropriate amount of PEDOT was introduced into the Metal‐Salen COF_EDA_, a narrower pore size (located at 1.7 nm) than the porosity of as‐synthesized Zn‐Salen COF_EDA_ is observed in the pore width distribution (Figure 5b). This indicates that in situ the PEDOT grows preferentially in the ordered 1D nanochannel.

**Figure 6:**
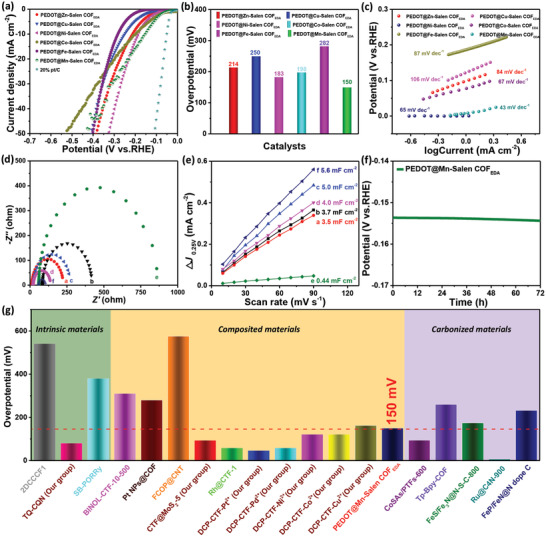
Electrochemical performance of PEDOT@Metal‐Salen COF_EDA_. a) LSVs of the catalysts in an H_2_SO_4_ aqueous solution (0.5 mol L^−1^, N_2_ saturated). b) Comparison of the performance of PEDOT@Metal‐Salen COF_EDA_ catalysts in this work. c) Tafel slopes of the PEDOT@Metal‐Salen COF_EDA_ electrocatalysts, calculated from the LSVs. d) Nyquist plots of PEDOT@Metal‐Salen COF_EDA._, and e) the capacitive current densities at 0.25 V as a function of scan rate for PEDOT@Metal‐Salen COF_EDA_ ((a) PEDOT@Zn‐Salen COF_EDA_, (b) PEDOT@Cu‐Salen COF_EDA_, (c) PEDOT@Ni‐Salen COF_EDA_, (d) PEDOT@Co‐Salen COF_EDA_, (e) PEDOT@Fe‐Salen COF_EDA_, (f) PEDOT@Mn‐Salen COF_EDA_). f) Chronoamperometry measurement for PEDOT@Mn‐Salen COF_EDA_ at a constant current density of 10 mA cm^−2^. g) HER performance of PEDOT@Mn‐Salen COF_EDA_ and other reported COFs.^[^ ^23^, ^24^, ^25^, ^44^, ^47^, ^48^, ^49^, ^50^, ^51^, ^52^, ^53^, ^54^, ^55^, ^56^ ^]^

The electrochemical performance of PEDOT@Metal‐Salen COF_EDA_ was analyzed as explained in this section. As shown in the polarization curves (**Figure** **6**a), introducing PEDOT into the nanochannels influences the performance of Metal‐Salen COF_EDA_, by shifting the overpotential to a more positive position and increasing the greater cathode current. When the current density is 10 mA cm^−2^, the overpotential of PEDOT@Zn, Cu, Ni, Co, and Fe‐Salen COF_EDA_ is ≈214, 250, 183, 198, 282, and 150 mV, respectively (Figure 6b). This excellent electrocatalytic performance may be attributed to the electrical conductivity and increased porosity caused by introducing the PEDOT (Figure 5b), which enhances the accessibility of electrons and H_ads_ at the metal active sites.

The Tafel slopes derived from the linear regions of the LSV curves were used to obtain a more detailed electrocatalytic mechanism. Compared with Metal‐Salen COF_EDA_, the PEDOT@Metal‐Salen COF_EDA_ shows a smaller Tafel slope, which indicates the faster electrocatalytic kinetics (Figure 6c). The rapid electrocatalytic kinetics may be explained as follows. i) The conductive linear macromolecule in the nanochannel can improve the conductivity of composites and transmit electrons to the metal active centers along the molecule cable. ii) The emerging porous structure enhances mass transportation of H_ads_ and the electrolyte by shortening the diffusion distance. PEDOT@Mn‐Salen COF_EDA_ can be used as an example: its Tafel slope (43 mV dec^−1^) is obviously less than that of Mn‐Salen COF_EDA_ (94 mV dec^−1^), shown in Figure 6c. This demonstrates that the HER mechanism of PEDOT@Mn‐Salen COF_EDA_ follows the Volmer–Heyrovsky process.

The EIS was conducted from 100 mHz to 100 kHz at open‐circuit voltage to describe the *R*_ct_. The Nyquist plots are presented in Figure 6d and the equivalent circuit diagram is shown in Figure S12 of the Supporting Information. As shown in Figure 6d, PEDOT@Metal‐Salen COF_EDA_ shows the semicircular behavior in the high frequency region. The Warburg impedance caused by diffusion in the low frequency region was not observed, which indicates that the electrochemical reaction controlled by the kinetics on the electrode surface is caused by rapid electron transfer in the electrolyte.^[^ ^46^ ^]^ The smaller the diameter of the semicircle, the faster the charge transfer at the electrode and electrolyte interface. It is clear that the *R*_ct_ of PEDOT@Metal‐Salen COF_EDA_ is lower than that of Metal‐Salen COF_EDA_, which confirms that PEDOT accelerates electron transfer.

Double layer capacitance (*C*_dl_) was also measured to determine the electrochemical active area (ECSA) of PEDOT@Metal‐Salen COF_EDA_ by CV in the non‐faradic region of 0.2–0.3 V versus reversible hydrogen electrode (RHE). As shown in Figure 6e and Figure S13 (Supporting Information), PEDOT@Mn‐Salen COF_EDA_ has the largest *C*_dl_ value (5.6 mF cm^−2^) compared to PEDOT@Zn, Cu, Ni, Co, and Fe‐Salen COF_EDA_. This confirms the more accessible ECSA, which is able to provide more reaction sites for H_ads_ adsorption. Figure 6f showed that PEDOT@Mn‐Salen COF_EDA_ maintain its potential for 72 h by means of chronoamperometry measurement, confirming the outstanding stability. Moreover, after the stability measurements, the SEM–EDS images of PEDOT@Mn‐Salen COF_EDA_ show the uniform distribution of elements (Figure S14, Supporting Information). In Figure S15 of the Supporting Information, compared with XPS analysis of Mn‐Salen COF_EDA_, there no obvious change is observed after HER in PEDOT@Mn‐Salen COF_EDA_. Based on the SEM–EDS and XPS analyses, it is speculated that the molecular structure of PEDOT@Mn‐Salen COF_EDA_ remains intact, which indicates that the electrocatalyst exhibited excellent stability during the catalysis. The electrocatalytic HER capacity of the reported COFs was also compared, as shown in Figure 6g. The results showed that PEDOT@Mn‐Salen COF_EDA_ exhibited outstanding performance.

## 3 Structure–Activity Relationship

**Figure 7:**
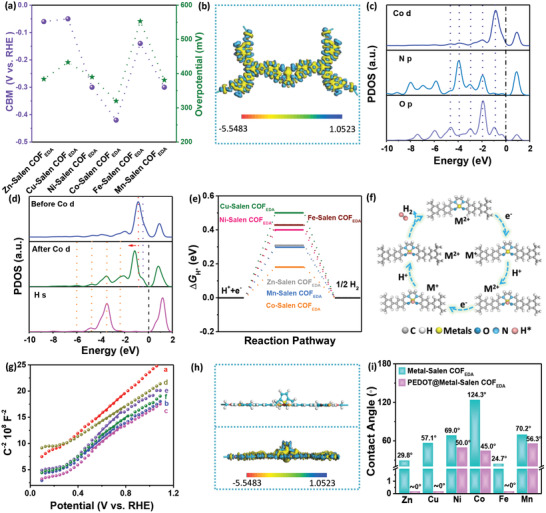
a) The relationship of between CBM and overpotential of Metal‐Salen COF_EDA_. b) The calculated charge density difference for the Co‐Salen COF_EDA_. c) The calculated PDOS of the Co, N, and O atom in Co‐Salen COF_EDA_. The black dashed line denotes the position of the Fermi level. d) Calculated PDOS of the Co atom before and after adsorption of H, and the H atom. e) Gibbs free energy for H_ads_ adsorption of Metal‐Salen COF_EDA_. f) Reaction mechanism for HER catalyzed by PEDOT@Metal‐Salen COF_EDA_. g) The Mott–Schottky plots of PEDOT@Metal‐Salen COF_EDA_ in an H_2_SO_4_ aqueous solution (0.5 mol L^−1^). h) The calculated charge density difference of the PEDOT@Mn‐Salen COF_EDA_. i) The contact angle of Metal‐Salen COF_EDA_ and PEDOT@Mn‐Salen COF_EDA_.

To demonstrate energy band modulation in Metal‐Salen COF_EDA_ by metal ion exchange, the band structures of different Metal‐Salen COF_EDA_ were studied using their Mott–Schottky plots to evaluate the influence of metal ions on HER performance. It is obvious that all Metal‐Salen COF_EDA_ have a positive slope and exhibit the n‐type semiconductor characteristic (Figure S15, Supporting Information). The flat potentials are estimated to be −0.06, −0.05, −0.30, −0.42, −0.14, and −0.31 V versus RHE for Zn, Cu, Ni, Co, Fe, and Mn‐Salen COF_EDA_. The conducting band minimum (CBM) of the n‐type semiconductor is considered to be about 0.10 eV below the flat band position, so the CBM positions are estimated to be −0.16, −0.15, −0.40, −0.52, −0.24, and −0.41 V versus RHE for Zn, Cu, Ni, Co, Fe, and Mn‐Salen COF_EDA_, which meets the requirements of water reduction.^[^ ^57^ ^]^ A correlation of the CBM position and the overpotential of Metal‐Salen COF_EDA_ is shown in **Figure** **7**a. With a decrease in the CBM position, the overpotential also decreases. It is possible that the more negative CBM of Co‐Salen COF_EDA_ provides more intense reducibility.^[^ ^30^ ^]^ According to the above analysis, the change in the coordination metal atoms can modulate the band structure of Salen COF_EDA_, thus optimizing HER performance.

In order to understand the effect of the electronic structure on electrochemical activity, theoretical calculations were done using Materials Studio. The structure models of Metal‐Salen COF_EDA_ and the detailed theoretical calculations (the DOS, charge density difference, and Δ*G*_H*_) are shown in Figures S17–S21 of the Supporting Information. The charge density difference was simulated to reveal the electronic structure. As shown in Figure S22 of the Supporting Information and Figure 7b, there is obvious electronic accumulation around the metals and electronic depletion around the N_2_–O_2_ moiety, which confirms the charge interaction between metals and COF_EDA_. This result is consistent with the XPS results obtained.

In order to further investigate the electronic structure of the metal centers, the partial density of states (PDOS) was calculated. For example, Co‐Salen COF_EDA_ showed that the PDOS of the *d*‐orbital near the Fermi level is larger than the *s*‐ and *p*‐orbital (Figure S23a, Supporting Information). This indicates a fast charge transfer that originates from the contribution of the *d*‐orbital.^[^ ^58^ ^]^ In addition, Figure 7c shows that the *d*‐orbital of Co matches the *p*‐orbital of N and O near the Fermi level; this demonstrates that the *d*‐orbital of Co participates in the formation of Co‐Salen motifs (N_2_–Co–O_2_). Compared with the *d*‐orbital of Co before absorption of H, the *d*‐orbital of Co after absorption of H shifts to a lower energy, i.e., below the Fermi level. Moreover, a new peak for the *d*‐orbital of Co is formed at the Fermi level. This analysis shows that the Co—H bonding orbital mainly consists of the *d*‐orbital of Co, which is well‐matched with the *s*‐orbital of absorbed H. For purposes of investigating the electrochemical reaction mechanism, the Δ*G*_H*_ of HER was simulated by adopting DFT. According to the calculated results (Figure 7e), the Co site has more appropriate free energy than Zn, Cu, Ni, Fe, and Mn in the Salen complexes. In short, by regulating the band and electronic structure of Metal‐Salen COF_EDA_‐based catalysts, the free energy of H_ads_ can be optimized to reduce the energy barrier.

According to the Tafel slope of PEDOT@Metal‐Salen COF_EDA_ (Figure 6c), it can be inferred that the hydrogen evolution process occurs through two steps. i) The proton‐coupled electron transfer contributes to generating the H_ads_ intermediate. ii) The second electron transfer of a proton and an electron occurs to produce H_2_, which is released from the active site. It then follows that the H_2_ evolution rate is positively correlated with the H^+^ and electron concentration. In other words, H^+^ and electron concentration are the essential factors that affect HER performance. Therefore, it is possible to facilitate the H_2_ evolution process by increasing proton and electron access to the active site; therefore, PEDOT was introduced into Metal‐Salen COF_EDA_ to generate heterostructures that were able to construct a highly active interface.

In order to reveal the origin of the excellent electrochemical activity of PEDOT@Metal‐Salen COF_EDA_, Mott–Schottky analysis was done to determine the charge carrier densities (*N*_d_) for HER in an acidic electrolyte. It is known that the slope of the Mott–Schottky plot is negatively correlated with *N*_d_; therefore, the smaller the slope of the Mott–Schottky plot, the larger the *N*_d_ and the faster the charge transfer kinetic during the HER process.^[^ ^59^ ^]^ Figure 7g shows that PEDOT@Mn‐Salen COF_EDA_ has the lowest slope compared to PEDOT@Zn, Cu, Ni, Co, and Fe‐Salen COF_EDA_. It indicates that PEDOT@Mn‐Salen COF_EDA_ has the largest number of charge carriers, which are responsible for improving the performance of HER.

The charge density differences of PEDOT@Mn‐Salen COF_EDA_ were also simulated. As shown in Figure 7h, electronic accumulation occurs in the interface between PEDOT and Mn‐Salen COF_EDA_, which indicates the facile charge interaction at the heterostructure of PEDOT and Mn‐Salen COF_EDA_. Interestingly, when analyzing the DOS, it was found that the electronic state of S in PEDOT is overlapped with Mn in Mn‐Salen COF_EDA_ (Figure S18b, Supporting Information). This proves that there is an interface interaction between PEDOT and Mn‐Salen COF_EDA_. In addition, the contact angle test demonstrated that the PEDOT@Metal‐Salen COF_EDA_ was more hydrophilic than Metal‐Salen COF_EDA_, which greatly increased the accessibility of the electrolyte to the active site (Figure 7i; Figure S24, Supporting Information).

Based on these results, the following opinions are proposed. i) The continuous band modulation and special coordination environment in Metal‐Salen COF_EDA_ allows the metal *d*‐orbital to interact better with the *s*‐orbital of H, which is conducive to electron transport and the HER process. ii) The 1D conductive macromolecule was introduced into the crystalline channel arrays of Metal‐Salen COF_EDA_ to form a heterostructure and reduce interfacial resistance to intramolecular charge transfer. Therefore, the heterostructure of PEDOT@Metal‐Salen COF_EDA_ is able to create interfacial active sites with high activity.

## 4 Conclusions

In summary, this study involved synthesizing a high crystalline Zn‐Salen COF_EDA_ through the condensation reaction of 1,3,5‐tris(4′‐hydroxy‐5′‐formylphenyl)benzene and ethylenediamine. The Zn^2+^ was exchanged with other metal ions for modulating the band structure in Zn‐Salen COF_EDA_. A series of Metal‐Salen COF_EDA_ were obtained (the metals included Cu, Ni, Co, Mn, and Fe). We also integrated conductive poly(3,4‐ethylenedioxythiophene) — with outstanding electrical characteristics, chemical stability, and hydrophilia—into the 1D channels of Metal‐Salen COFs to improve HER performance by means of in situ solid‐state polymerization. Finally, the Metal‐Salen COF_EDA_ and PEDOT@Metal‐Salen COF_EDA_ obtained as electrocatalysts of HER were evaluated. The PEDOT@Mn‐Salen COF_EDA_ displayed an overpotential of 150 mV and a Tafel slope of 43 mV dec^−1^. This is an excellent electrochemical performance for COF‐based HER electrocatalysts.

The following conclusions can be drawn. i) The permanent porosity and *π*‐conjugated framework facilitate the diffusion of reactants and electron migration. ii) The electron‐donating N_2_–O_2_ binding motifs and the electron‐withdrawing metal unoccupied orbital construct a D–A type semiconductor, and the generated electronic push–pull effect is beneficial in promoting exciton separation and stimulating the HER process. iii) Modulation of the energy band structure in Metal‐Salen COF_EDA_ results in the metal *d*‐orbital interacting better with the s‐orbital of H, which is conducive to electron transport and HER. iv) Introducing PEDOT into the 1D channel of Metal‐Salen COF_EDA_ improves conductivity and leads to the construction of highly active interfacial sites.

## Conflict of Interest

The authors declare no conflict of interest.

## Supporting information

Supporting Information

## Data Availability

Research data are not shared.
