# Supplementary material for: Modulating the Band Structure of Metal Coordinated Salen COFs and an In Situ Constructed Charge Transfer Heterostructure for Electrocatalysis Hydrogen Evolution
Source: Adv Sci (Weinh). 2022 Jun 3;9(22):2105912. doi: 10.1002/advs.202105912 (PMC9353467; doi:10.1002/advs.202105912)
Supplement: Supplementary file 1 — Supporting Information [file ADVS-9-2105912-s001.pdf]

Supporting Information

Modulating the Band Structure of Metal Coordinated Salen COFs and an *in-situ* Constructed Charge Transfer Heterostructure for Electrocatalysis Hydrogen Evolution

Boying Zhang, Liling Chen, Zhenni Zhang, Qing Li, Phathutshedzo Khangale, Diane Hildebrandt, Xinying Liu,* Qingliang Feng, Shanlin Qiao**

B. Zhang, L. Chen, Z. Zhang, Q. Li, S. Qiao

College of Chemistry and Pharmaceutical Engineering, Hebei University of Science and Technology, Shijiazhuang 050018, China

E-mail: ccpeslqiao@hebust.edu.cn (S. Qiao)

iccaslq@hebust.edu.cn (Q. Li)

B. Zhang, P. Khangale

Department of Chemical Engineering, Faculty of Engineering and the Built Environment, University of Johannesburg, Doornfontein 2028, South Africa

D. Hildebrandt

African Energy Leadership Centre, WITS Business School & Molecular Science Institute, School of Chemistry, University of Witwatersrand, Johannesburg 2050, South Africa

X. Liu

Institute for Development of Energy for African Sustainability, University of South Africa, Florida 1709, South Africa

E-mail: liux@unisa.ac.za (X. Liu)

L. Chen, Q. Feng

Key Laboratory of Special Functional and Smart Polymer Materials of Ministry of Industry and Information Technology School of Chemistry and Chemical Engineering, Northwestern Polytechnical University, Xi'an 710072, China

S. Qiao

Hebei Electronic Organic Chemicals Technology Innovation Center, Shijiazhuang 050018, China

E-mail: ccpeslqiao@hebust.edu.cn (S. Qiao)

Table of Contents

Section S1. Synthesis of Metal-Salen COF_{EDA} and PEDOT@Metal-Salen COF_{EDA}

Section S2. Supplementary Figures

Figure S1. *Synthesis of 1,3,5-tris(4-methoxy-5-formylphenyl)benzene.*

Figure S2. *Synthesis of 1,3,5-tris(4-hydroxy-5-formylphenyl)benzene.*

Figure S3. *SEM images of Zn-Salen* COF_{EDA}.

Figure S4. *SEM images and EDS element mapping images of Metal-Salen* COF_{EDA}.

Figure S5. *XPS spectra of Metal-Salen* COF_{EDA}.

Figure S6. *N 1s, O 1s and Zn 2p high resolution XPS spectra of Metal-Salen* COF_{EDA}.

Figure S7. *Stability measurement of Co-Salen* COF_{EDA} *in a solution of aqueous* H_2SO_4 *(0.5 mol* L^{-1}*).*

Figure S8. *TEM image of Co-Salen* COF_{EDA} *after the stability test.*

Figure S9. *High-resolution (a) Co 2p, (b) N 1s, (c) O 1s spectra of Co-Salen* COF_{EDA}. *(d, g, j) Co 2p, (e, h, k) N 1s, (f, i, l) O 1s spectra of Co-Salen* COF_{EDA} *after HER stability testing (three parallel tests).*

Figure S10. *(a) FT-IR spectra Zn-Salen* COF_{EDA}*, PEDOT@Metal-Salen* COF_{EDA}*. (b) PXRD patterns of PEDOT@M-Salen* COF_{EDA}.

Figure S11. *SEM images and EDS element mapping images of: (a) PEDOT@Zn-Salen* COF_{EDA}*; (b) PEDOT@Cu-Salen* COF_{EDA}*; (c) PEDOT@Ni-Salen* COF_{EDA}*; (d) PEDOT@Co-Salen* COF_{EDA}*; (e) PEDOT@Fe-Salen* COF_{EDA}*; (f) PEDOT@Mn-Salen* COF_{EDA}.

Figure S12. *Equivalent circuit diagram.*

Figure S13. *Cyclic voltammetry curves of PEDOT@Metal-Salen* COF_{EDA} *in the region of 0.2–0.30 V vs. RHE.*

Figure S14. *SEM and EDS images of PEDOT@Mn-Salen* COF_{EDA} *after the stability test.*

Figure S15. *High-resolution (a) Mn 2p, (b) N 1s, (c) O 1s spectra of Mn-Salen* COF_{EDA}*. (d, g, j) Mn 2p, (e, h, k) N 1s, (f, i, l) O 1s spectra of PEDOT@Mn-Salen* COF_{EDA} *after HER stability testing (three parallel tests).*

Figure S16. *Mott-Schottky (M–S) plots for: (a) PEDOT@Zn-Salen* COF_{EDA}*; (b) PEDOT@Cu-Salen* $COFE_{DA}$*; (c) PEDOT@Ni-Salen* COF_{EDA}*; (d) PEDOT@Co-Salen* COF_{EDA}*; (e) PEDOT@Fe-Salen* COF_{EDA}*; (f) PEDOT@Mn-Salen* COF_{EDA}*. Measured in 0.2 M* Na_2SO_4 *with Ag/AgCl (+0.197 V vs NHE) as the reference electrode.*

Section S3. Theoretical Calculations

Figure S17. *Proposed models for Metal-Salen* COF_{EDA} *for calculating DOS, charge density difference.*

Figure S18. *Models forMetal-Salen* COF_{EDA} *after adsorbing the H atom.*

Figure S19. *(a) Optimized geometric structure of the 2 × 2 × 1 supercell of Salen* COF_{EDA}*. (b) Three-dimensional view of Salen* COF_{EDA}*. (c) Geometric structure of simplified model catalysts (H atom: white, C atom: cyan, N atom: read, O atom: yellow, Zn atom: grey, Cu atom: green, Ni atom: pink, Co atom: orange, Fe atom: light blue, Mn atom: wine red).*

Figure S20. *Geometric structure after cluster calculations.*

Figure S21. *Adsorbed H geometric structure after cluster calculations.*

Figure S22. *Calculated charge density difference for the Zn-Salen* COF_{EDA}*, Cu-Salen* COF_{EDA}*, Ni-Salen* COF_{EDA}*, Fe-Salen* COF_{EDA} *and Mn-Salen* COF_{EDA}*.*

Figure S23. *Calculated PDOS of: (a) Co atom in Co-Salen* COF_{EDA} *(the black dashed line denotes the position of the Fermi level); (b) PEDOT@Mn-Salen* COF_{EDA}*.*

Figure S24. *Contact angle of Metal-Salen* COF_{EDA} *and PEDOT@Mn-Salen* COF_{EDA}*.*

Table S1. *Fractional atomic coordinates for the unit cell of Salen* COF_{EDA}*.*

Section S4. References

Section S1. Synthesis of Metal-Salen COF_{EDA} and PEDOT@Metal-Salen COF_{EDA}

1.1 Materials

5-Bromo-2-methoxybenzaldehy was purchased from Shanghai Yien Chemical Technology Co., Ltd. 1,3,5-Tris(4,4,5,5-teyramethyl-1,3.2-dioxaborolan-2-yl)benzene was obtained from Shanghai Bide Medical Technology Co., Ltd. Ethanediamine, dichloromethane, ethyl acetate, normal hexane, petroleum ether, N.N-dimethylformamide, ethanol, methanol and acetone were purchased from Damao chemical reagent factory (Tianjin, China). Mesitylene, 2,5-dibromo-3.4-ethylenedioxythiophene $Zn(OAC)_2 \cdot 2H_2O$, $Cu(OAC)_2 \cdot H_2O$, $Ni(OAC)_2 \cdot 4H_2O$, $Co(OAC)_2 \cdot 2H_2O$, $Fe(OAC)_2 \cdot H_2O$, and $Mn(OAC)_2 \cdot 4H_2O$ were obtained from Aladdin Industrial Corporation (Shanghai, China). All chemicals were used without further purification.

1.2 Instrumental characterization

The PXRD data were collected on a SmartLab9KW diffractometer (Rigaku, Cu Ka). The solid UV spectra were recorded using a UV–vis Spectrometer Lambda 750S (Perkin Elmer, Inc., USA) in the range of 200–800 nm at room temperature. Fourier transform infrared (FT–IR) spectra were recorded using a Thermo Scientific Nicolet iS10 spectrometer. Solid ^{13}C NMR experiments were characterized using a Bruker 400 MHz. A scanning electron microscope (SEM, JEOL) equipped with an energy–dispersive spectrometer recorded the morphology of the samples. TEM and HRTEM images were recorded on a transmission electron microscope (JEOL, JEM–2100). Nitrogen adsorption/desorption isotherms were obtained on a Quantachrome Autosorb iQ apparatus at 77 K. The specific surface areas were calculated using the Brunauer–Emmett–Teller (BET) method. The samples were degassed at 150 °C for 12 h before measurements were taken. The XPS data were collected using a Thermo Scientific K-Alpha spectrometer.

Electrochemical measurements were performed using three–electrodes on an electrochemical workstation (Princeton, U.S.). The three-electrode setup comprised a

working, counter and reference electrode, which were a glass carbon electrode (4 mm in diameter) coated with catalyst, a graphite rod, and Ag/AgCl (sat. KCl). The electrocatalytic performance of the electrocatalysts were tested in an N_2–saturated aqueous solution of H_2SO_4 (0.5 mol L^{-1}).

In LSV measured reaction current cannot reflect the intrinsic behavior of electrocatalyst due to ohmic resistance effect. So, resistance test was made for iR-compensation of all initial data for further analysis. All the potentials were converted to the reversible hydrogen electrode (RHE) using the following formula:

$$E_{\mathrm{RHE}} = E_{\mathrm{Ag/AgCl}} + 0.197\ \mathrm{V} + 0.0591 * \mathrm{pH} - iRs \qquad \text{Equation (1)}$$

The scan rate of the linear sweep voltammetry (LSV) was 5 mV s^{-1}. Electrochemical impedance spectroscopy (EIS) was measured at a frequency from 100 kHz to 100 mHz and an AC voltage of 5 mV. The double layer capacitance (C_{dl}) was obtained by cyclic voltammetry (CV) under the potential windows of 0.00–0.20 V vs. RHE with scan rates of 20, 40, 60, 80 and 100 mV s^{-1}. The differences in current density variation ($\Delta J = J_a – J_c$, where J_a and J_c are the anodic and cathodic current, respectively) at an overpotential of 0.10 V plotted against the scan rate and fitted to a linear regression enabled estimation of the C_{dl} for the electrocatalysts.

The catalyst ink solutions were prepared by adding 4 mg of each catalyst and 30 μL of 5 wt % Nafion to a 1mL water/ethanol (V/V=3:1) mixture solution. The mixed suspensions were ultrasonicated for 1 h. Then, 5 μL of each catalyst ink was uniformly dispersed on the polished glass carbon electrode and dried at room temperature. The catalyst loading on the glass carbon electrode was about 0.159 mg cm^{-2}.

1.3 Synthesis of 1,3,5-tris(4-methoxy-5-formylphenyl)benzene

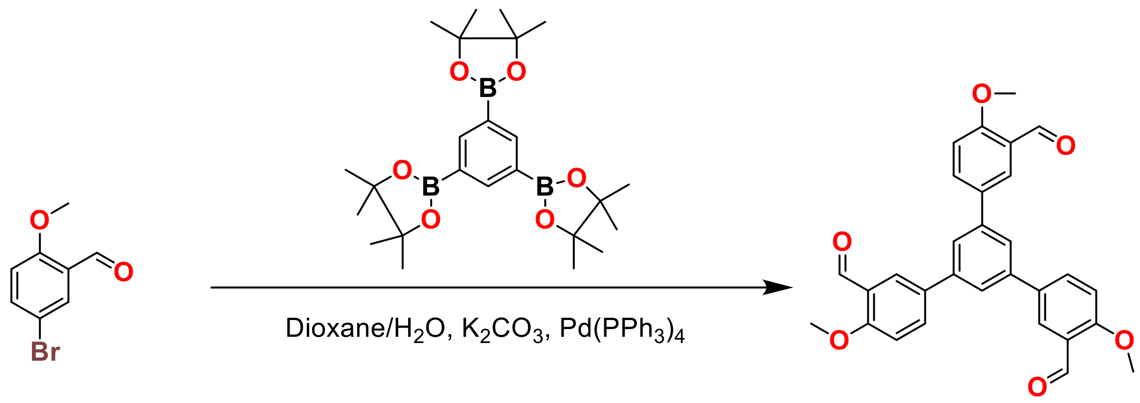

Figure S1. Synthesis of 1,3,5-tris(4-methoxy-5-formylphenyl)benzene.

1,3,5-Tris(4-methoxy-5-formylphenyl)benzene was synthesized according to the procedure described in the literature.[1] 5-Bromo-2-methoxybenzaldehy (2.5 g, 10 mmol), 1,3,5-tris(4,4,5,5-tetramethyl-1,3,2-dioxaborolan-2-yl)benzene (1.50 g, 3.33 mmol), K_2CO_3 (3.3 g, 24 mmol) and $Pd(PPh_3)_4$ (0.23 g, 0.2 mmol) in dioxane/H_2O (3/1 v/v, 80 mL) were degassed for 10 min. The suspension was stirred under N_2 at 100 °C for 24 h. After cooling to room temperature, the mixture was concentrated and then extracted with dichloromethane. The organic phase was dried over anhydrous Na_2SO_4 and then concentrated under reduced pressure to remove the solvent. The crude product was purified by silica gel column chromatography (hexanes/ethyl acetate (3:1 v/v) to obtain 1,3,5-tris(4-methoxy-5-formylphenyl)benzene. ^{1}H NMR (400 MHz, $CDCl_3$): δ 4.05 (s, 9H, OCH_3), 7.15 (d, *J*=8.4 Hz, 3H), 7.74 (s, 3H), 7.93 (dd, *J*=2.8, 2.4 Hz, 3H), 8.17 (d, *J*=2.0 Hz, 3H), 10.57 (s, 3H, CHO).

1.4 *Synthesis of 1,3,5-tris(4-hydroxy-5-formylphenyl)benzene*

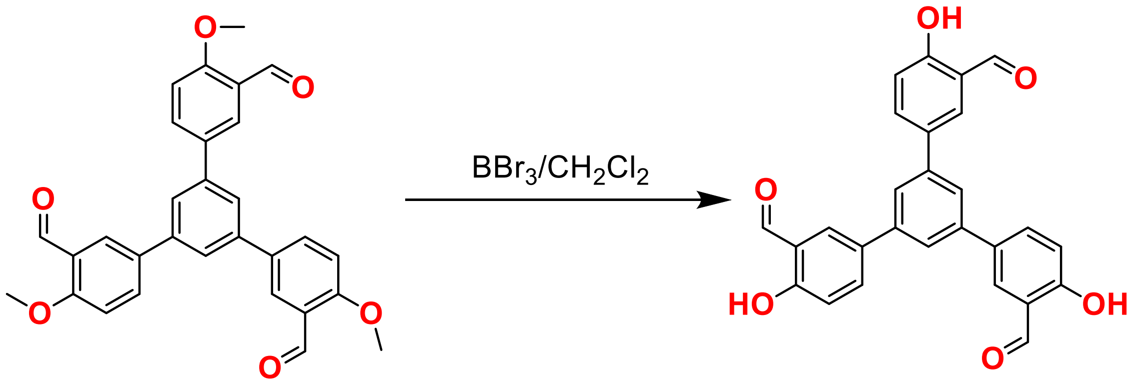

Figure S2. Synthesis of 1,3,5-tris(4-hydroxy-5-formylphenyl)benzene.

1,3,5-Tris(4-hydroxy-5-formylphenyl)benzene was synthesized according to the procedure described in the literature.[2] BBr_3 (1 M in CH_2Cl_2, 3.2 mL, 3.2 mmol) was added at –78 °C under nitrogen to a suspension of 1,3,5-tris(4-methoxy-5-formylphenyl)benzene (0.3 g, 0.63 mmol) in dry CH_2Cl_2 (6 mL). After 10 min, the external temperature was raised to –15 °C, and the dark brown suspension was stirred for 1 h. The resulting mixture was slowly poured into chilled water (10 mL) and stirred continuously until two liquid layers were formed. The aqueous phase was extracted with Et_2O (3*20 mL). The combined organic layers were washed with brine, dried with $MgSO_4$, and concentrated under reduced pressure. Purification by column chromatography (CC) (silica gel, CH_2Cl_2/MeOH=100/1, by vol.) delivered the title compound as a pale white solid. ^{1}H NMR (400MHz, DMSO): δ 7.15 (d, *J*=8.4 Hz, 3H),7.81 (s, 3H), 8.07 (d, *J*=9.6 Hz, 3H), 8.11 (s, 3H), 10.35 (s, 3H, CHO), 10.93 (s, 3H, OH).

1.5 Synthesis of Zn-Salen COF_{EDA}

1,3,5-tris(4'-hydroxy-5'-formylphenyl)benzene (THB) (0.03 mmol, 13.2 mg) and $Zn(OAC)_2 \cdot 2H_2O$ (14.8 mg) was weighted into a Pyrex tube (volume of ca. 10 mL). The mixture was dissolved in 1.5 mL of mesitylene/EtOH (1:1 v/v) and sonicated for 5 mins. Then ethanediamine (0.045 mmol, 5 μL) was added to the mixture. The mixed solution was ultrasonicated for another 2 min. After the aqueous acetic acid (6 M, 0.15 mL) was added, the solution was sonicated for 5 mins to ensure uniform dispersion. The Pyrex tube was degassed by means of three freeze-pump-thaw cycles and flame-sealed. The tube was placed in an oven at 120 °C for 3 days. When the reaction time was up, the ampoule was cooled to room temperature and opened. The product was collected centrifugally and cleaned with DMF, ethanol and acetone. The powder was dried in an oven at 100 °C under vacuum overnight.

1.6 Synthesis of Metal-Salen COF_{EDA}

The Zn-Salen COF_{EDA} and $M(OAc)_2 \cdot nH_2O$ were weighted into a glass vial, and dry ethyl alcohol was added. The mixture was continuously stirred at room temperature for 48 hours, with the solution being refreshed three times. The solid was collected by centrifugation and washed with ethyl alcohol and acetone several times. The powder was dried in an oven at 80 °C under vacuum overnight.

1.7 Synthesis of PEDOT@Metal-Salen COF_{EDA}

PEDOT@Metal-Salen COF_{EDA} was synthesized according to the procedure described in the literature.[3] 2,5-dibromo-3,4-ethylenedioxythiophene (DBrEDOT, 20 mg) was dissolved using acetone (15 mL) in a glass vial. The Metal-Salen COF_{EDA} powder (200 mg) was added to the mixture containing DBrEDOT. The mixture was continuously stirred at room temperature for 1 h. The solid was collected by centrifugation and washed with hexane to remove the DBrEDOT on the outer surface of the Metal-Salen COF_{EDA}. The powder was dried in an oven at room temperature under vacuum overnight. The glass vial containing the powder was then sealed under N_2 and heated at 60 °C for 3 days, and then at 85 °C for 1 day. The powder was then washed with acetone and dried in an oven at 100 °C under vacuum overnight.

Calculation mass ratio of Zn-Salen COF_{EDA} to PEDOT based on XPS analysis.

1) Results from structure:

a. Chemical formula for unit cell of Zn-Salen COF_{EDA}:

$C_{60}H_{42}N_6O_6Zn_3$ (Molecular Weight: 1139.25)

b. Chemical formula for repeating unit of PEDOT:

$C_6H_4O_2S$ (Molecular Weight: 140.16)

2) Results from XPS elemental analysis:

C (61.61 wt%), H (3.65 wt%), O (9.74 wt%), N (6.71 wt%), S (2.08 wt%), Zn (15.65 wt%)

3) Atomic mass (m_a) from periodic table of elements:

C: 12.01; H: 1.01; N: 14.01; S: 32.06; O: 16.00; Zn: 65.39

4) Calculate the molar ratio of N: S

$$(6.71\ wt\%/14.01) : (2.08\ wt\%/32.06) = 7.39:1$$

5) Calculate the molar ratio of Unit cell of Zn-Salen COF_{EDA} ($C_{60}H_{42}N_6O_6Zn_3$): Repeating unit of PEDOT($C_6H_4O_2S$)

$$(7.39/6):1 = 1.23:1$$

6) Calculate the mass ratio of Zn-Salen COF_{EDA}: PEDOT

$$(1139.25\times1.23): 140.16 \approx 10:1$$

Section S2. Supplementary Figures

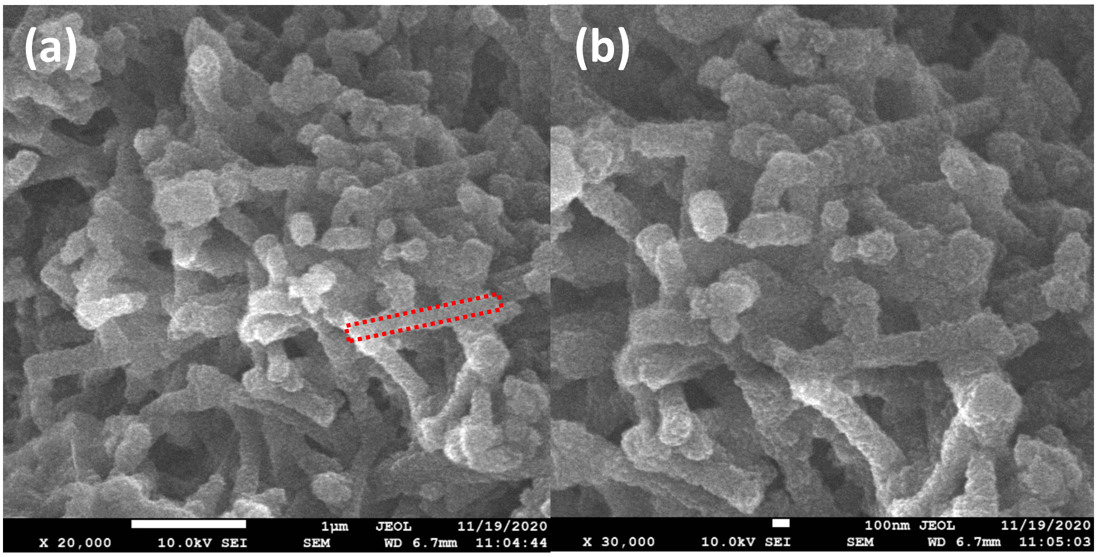

Figure S3. SEM images of Zn-Salen COF_{EDA}.

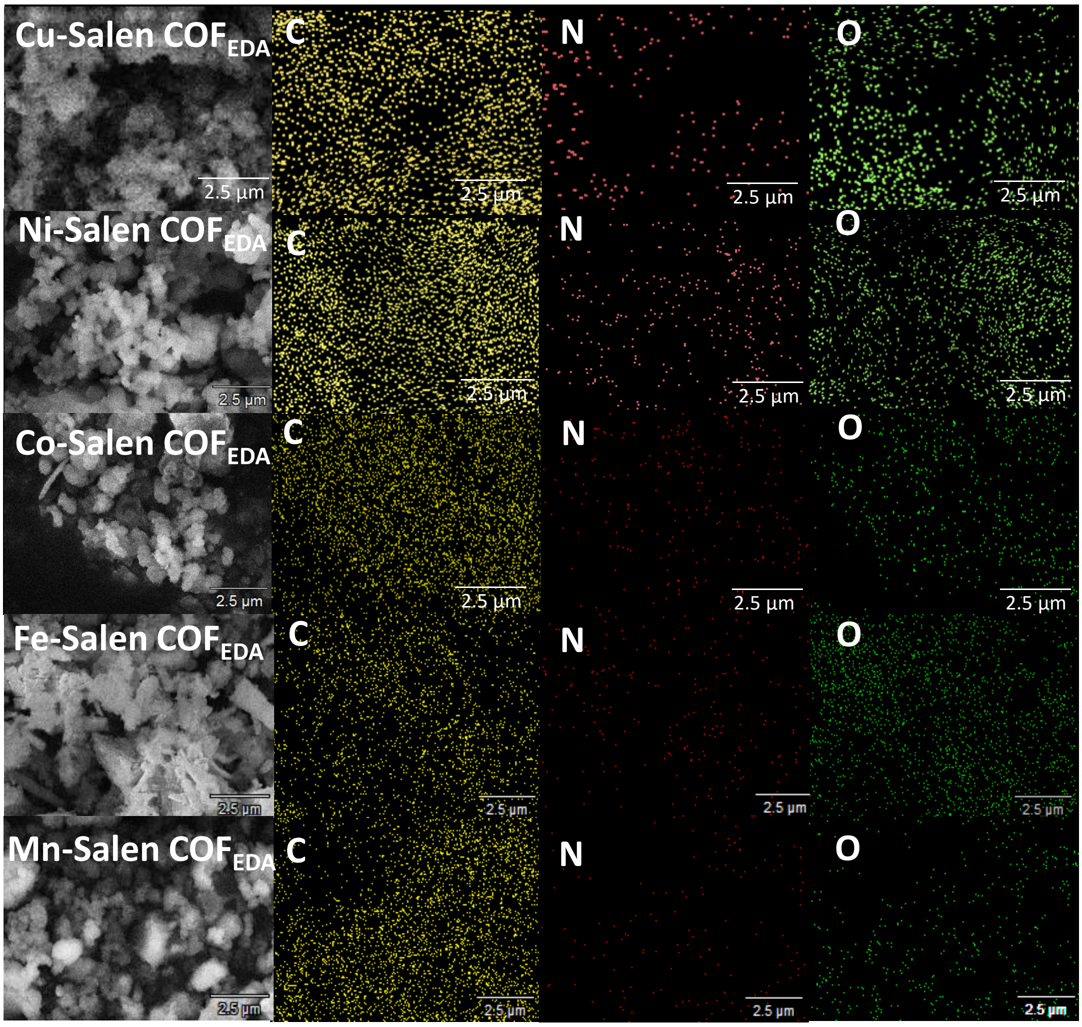

Figure S4. SEM images and EDS element mapping images of Metal-Salen COF$_{EDA}$.

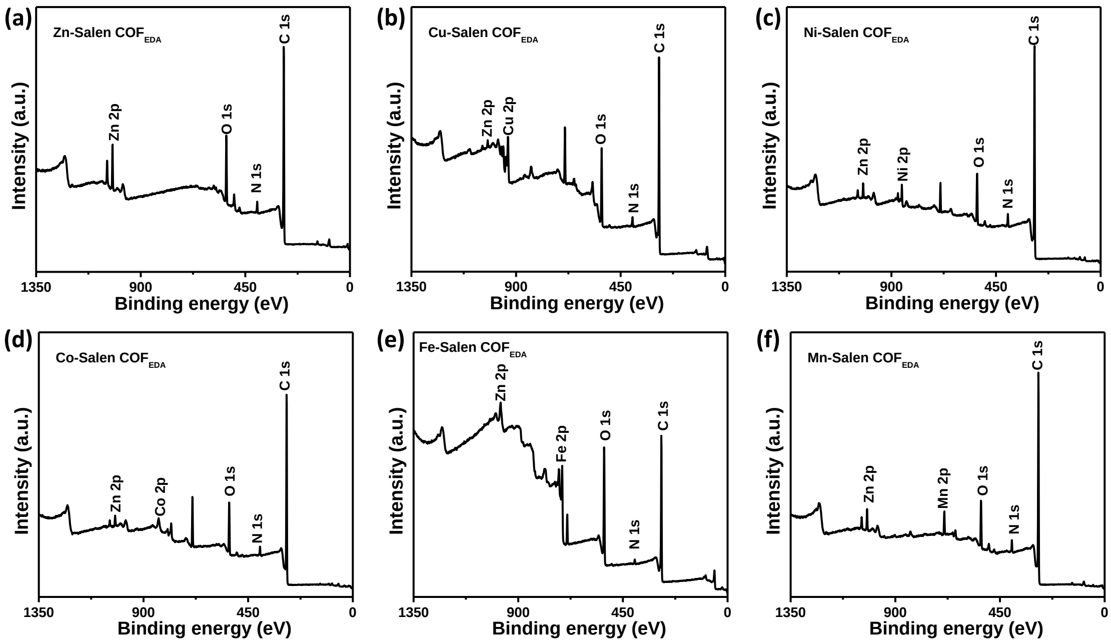

Figure S5. XPS spectra of Metal-Salen COF$_{EDA}$.

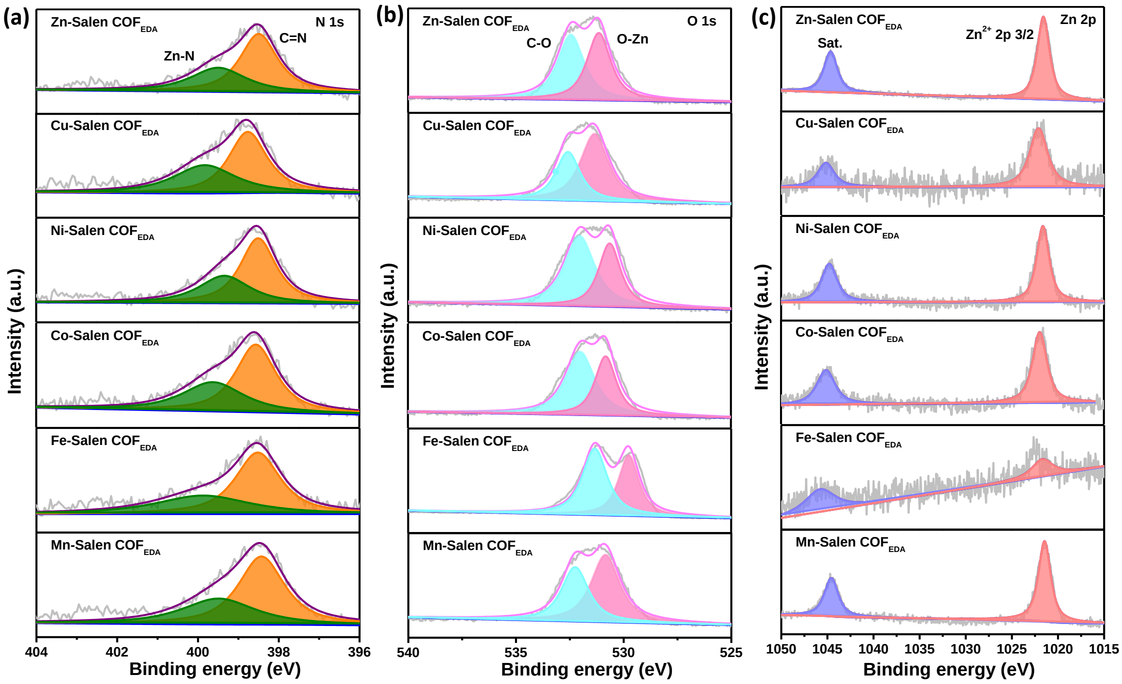

Figure S6. N 1s, O 1s and Zn 2p high resolution XPS spectra of Metal-Salen COF_{EDA}.

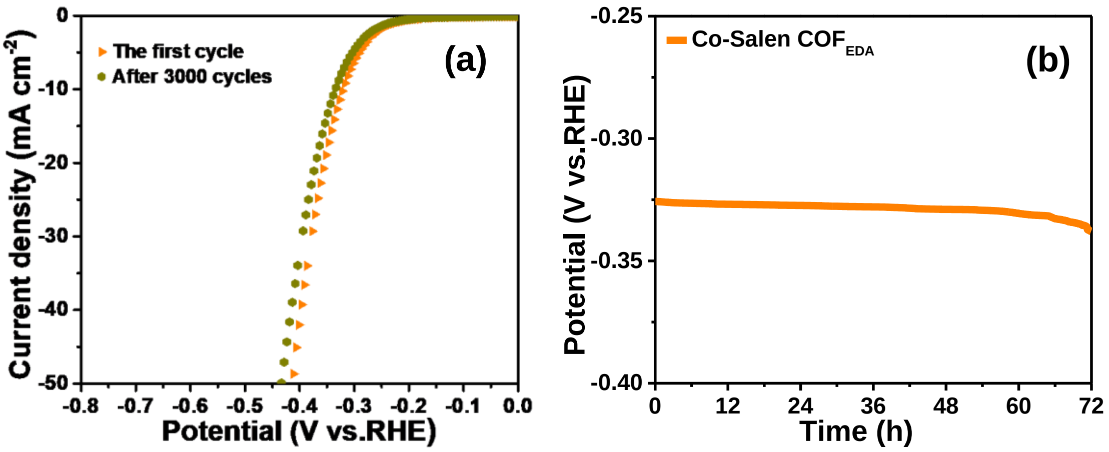

Figure S7. Stability measurement of Co-Salen COF_{EDA} in a solution of aqueous H_2SO_4 (0.5 mol L^{-1}). (a) Cyclic voltammetry stability for Co-Salen COF_{EDA}. (b) Chronopotentiometric stability test for Co-Salen COF_{EDA} at –10 mA cm^{-2}.

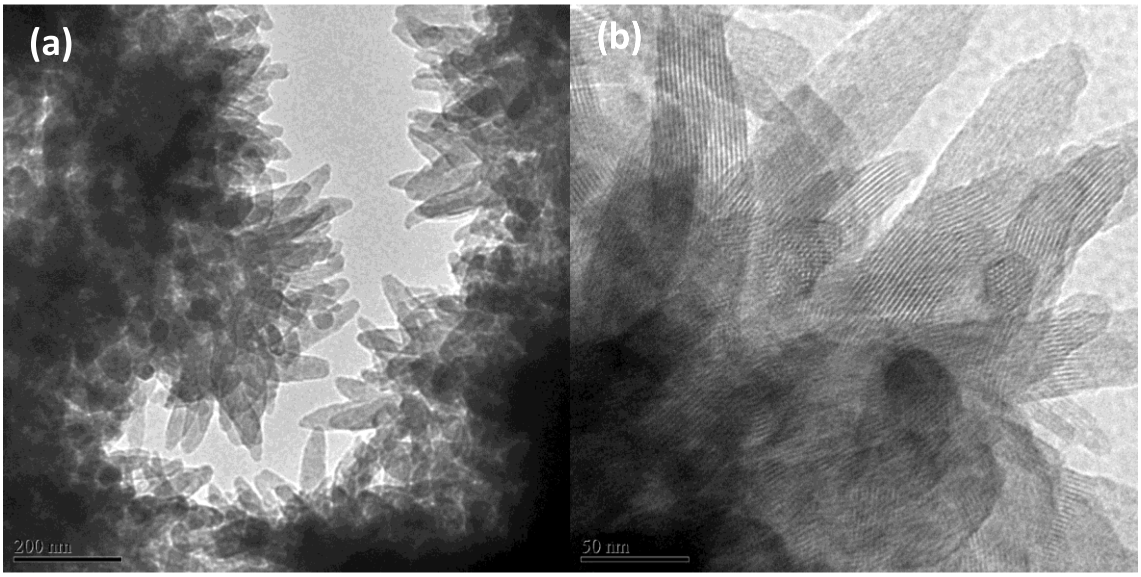

Figure S8. TEM image of Co-Salen COF_{EDA} after the stability test.

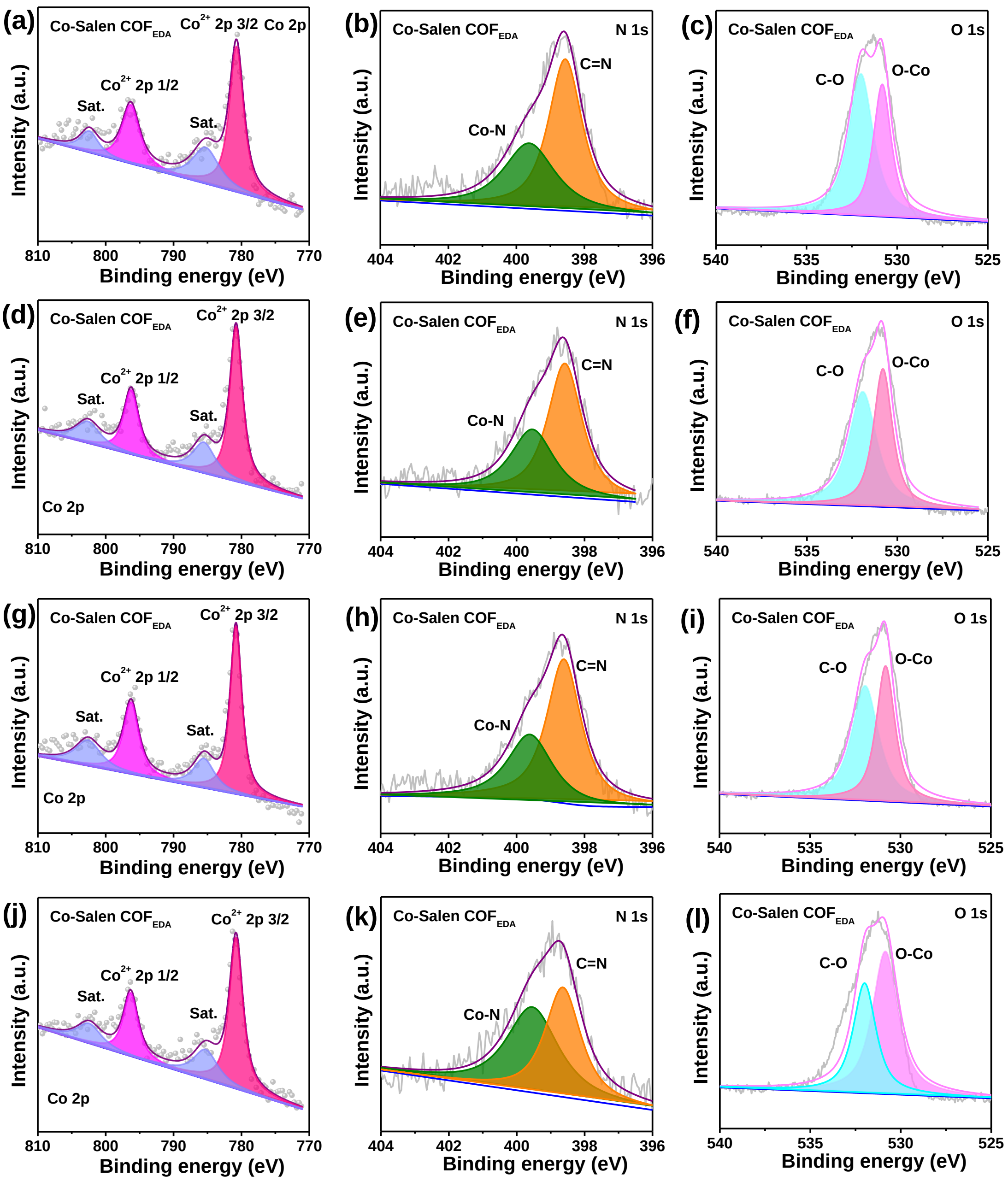

Figure S9. High-resolution (a) Co 2p, (b) N 1s, (c) O 1s spectra of Co-Salen COF_{EDA}. (d, g, j) Co 2p, (e, h, k) N 1s, (f, i, l) O 1s spectra of Co-Salen COF_{EDA} after HER stability testing (three parallel tests).

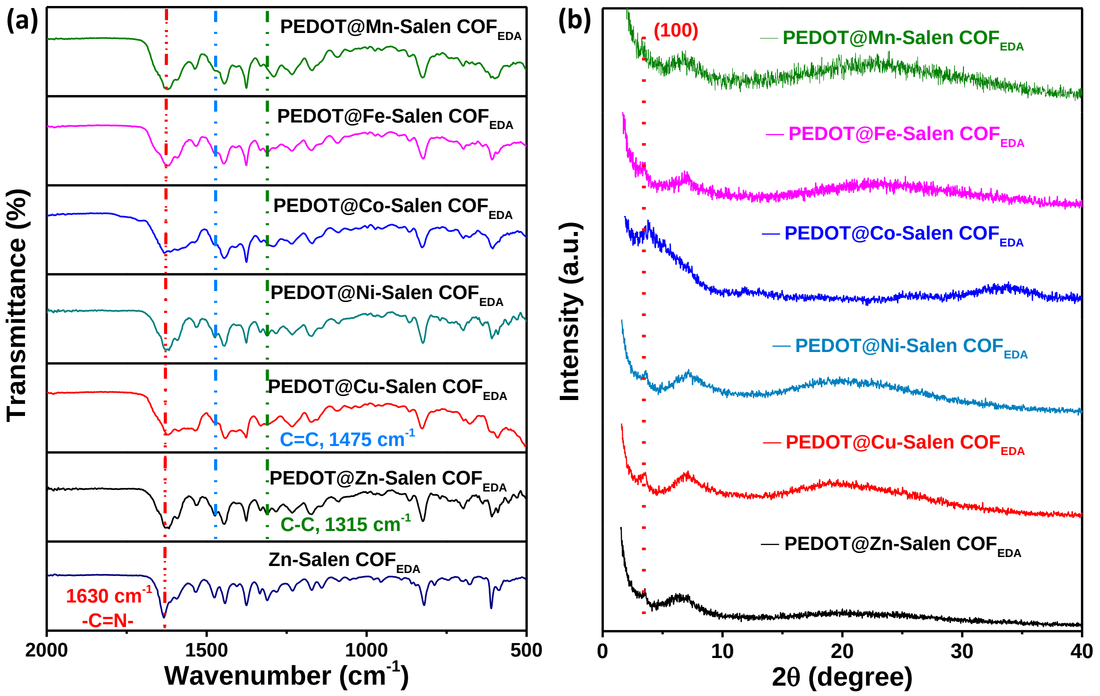

Figure S10. (a) FT-IR spectra Zn-Salen COF$_{EDA}$, PEDOT@Metal-Salen COF$_{EDA}$. (b) PXRD patterns of PEDOT@M-Salen COF$_{EDA}$.

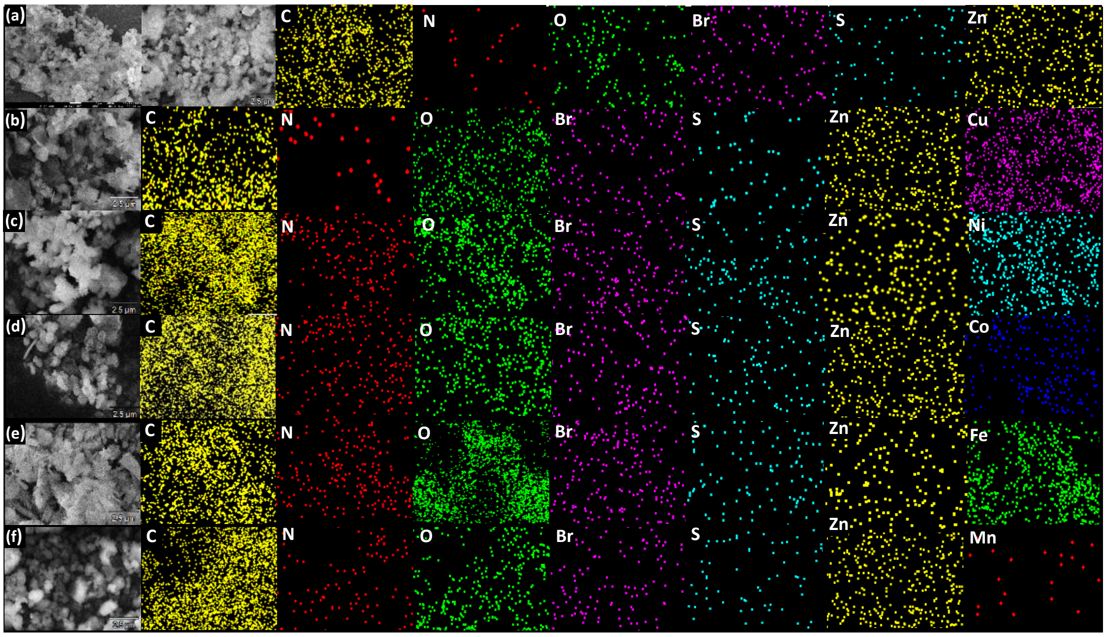

Figure S11. SEM images and EDS element mapping images of: (a) PEDOT@Zn-Salen COF_{EDA}; (b) PEDOT@Cu-Salen COF_{EDA}; (c) PEDOT@Ni-Salen COF_{EDA}; (d) PEDOT@Co-Salen COF_{EDA}; (e) PEDOT@Fe-Salen COF_{EDA}; (f) PEDOT@Mn-Salen COF_{EDA}.

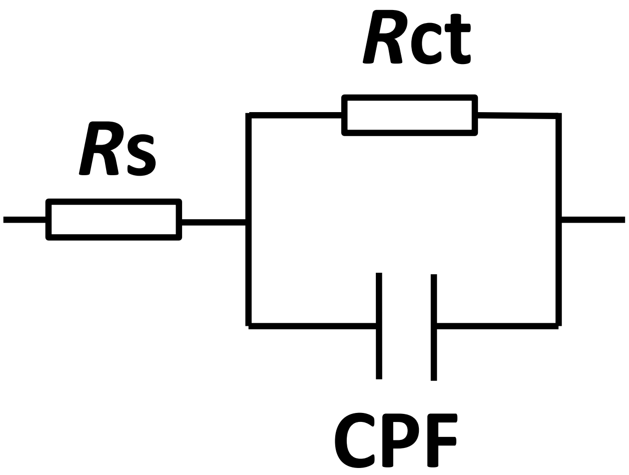

Figure S12. Equivalent circuit diagram.

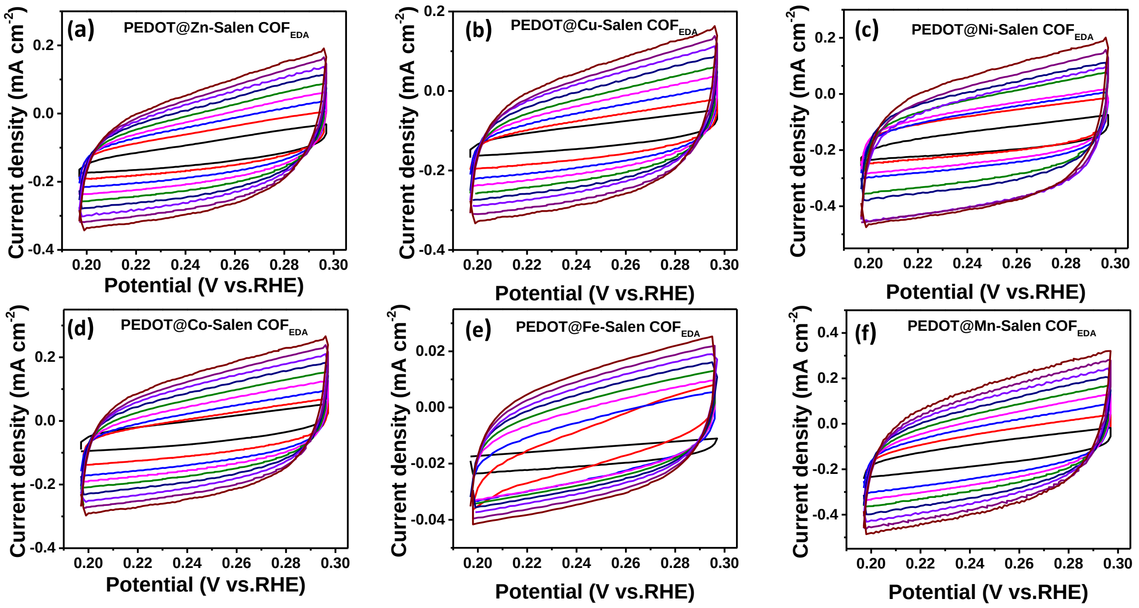

Figure S13. Cyclic voltammetry curves of PEDOT@Metal-Salen COF$_{EDA}$ in the region of 0.2–0.30 V vs. RHE.

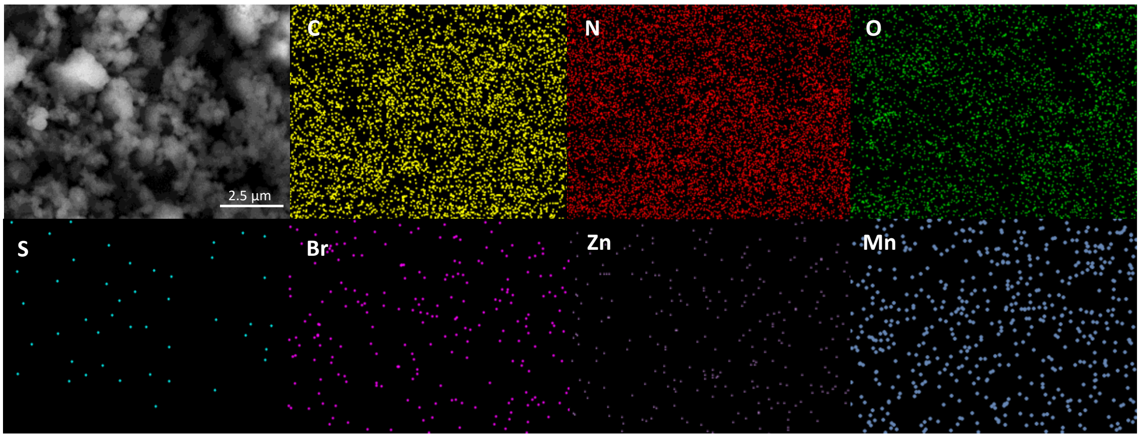

Figure S14. SEM and EDS images of PEDOT@Mn-Salen COF_{EDA} after the stability test.

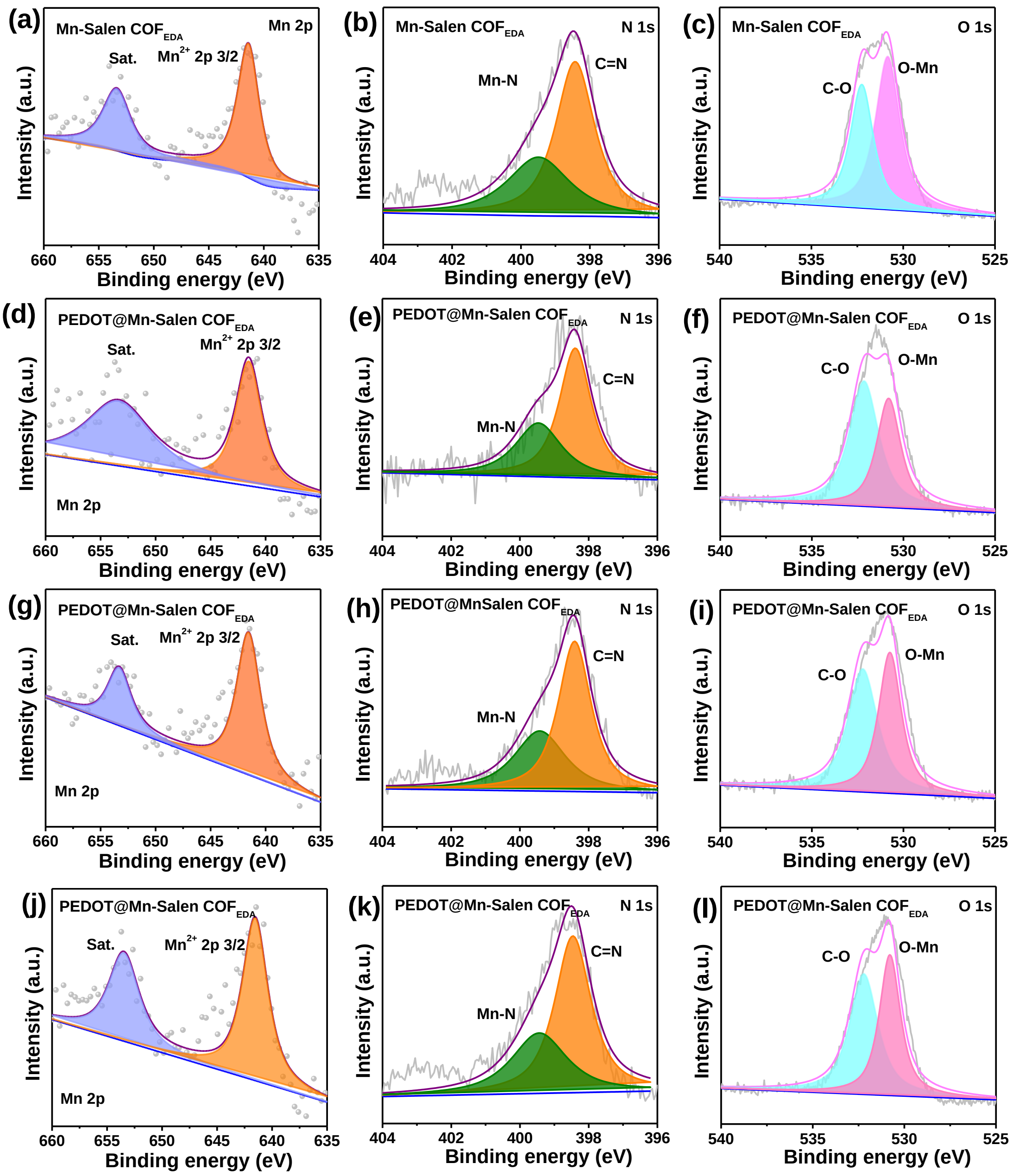

Figure S15. High-resolution (a) Mn 2p, (b) N 1s, (c) O 1s spectra of Mn-Salen COF_{EDA}. (d, g, j) Mn 2p, (e, h, k) N 1s, (f, i, l) O 1s spectra of PEDOT@Mn-Salen COF_{EDA} after HER stability testing (three parallel tests).

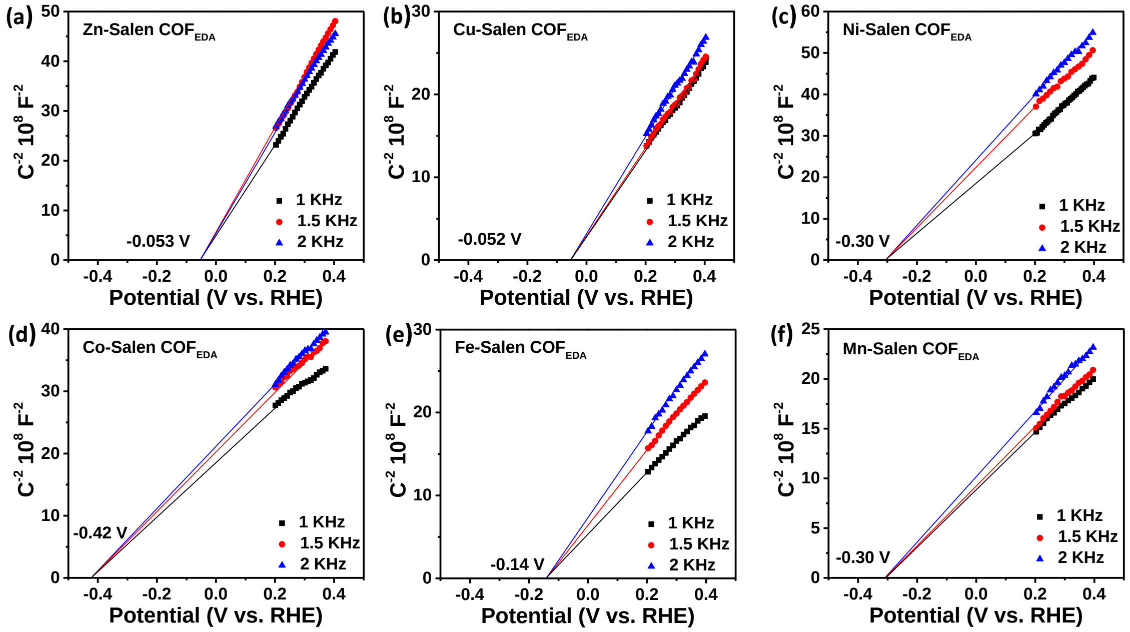

Figure S16. Mott-Schottky (M-S) plots for: (a) PEDOT@Zn-Salen COF$_{EDA}$; (b) PEDOT@Cu-Salen COF$_{EDA}$; (c) PEDOT@Ni-Salen COF$_{EDA}$; (d) PEDOT@Co-Salen COF$_{EDA}$; (e) PEDOT@Fe-Salen COF$_{EDA}$; (f) PEDOT@Mn-Salen COF$_{EDA}$. Measured in 0.2 M Na_2SO_4 with Ag/AgCl (+0.197 V vs NHE) as the reference electrode.

Section 3. Structure simulation and theoretical calculations

Molecular modeling of these COFs was generated with the Materials Studio (ver. 2019) suite of programs. The lattice models (e.g., cell parameters, atomic positions, and total energies) were fully optimized using MS Forcite molecular dynamics module method. Finally, Pawley refinement was carried out using Reflex, a software package for crystal determination from PXRD pattern. Unit cell dimension was set to the theoretical parameters. The Pawley refinement was performed to optimize the lattice parameters iteratively until the R_{wp} value converges and the overlay of the observed with refined profiles shows good agreement.

The density of states (DOS) and charge density difference were calculated by means of Material Studio, using the *Dmol 3* module. The Perdew-Burke-Ernzerhof functional of GGA was employed in cell relaxation and geometry optimization. DNP basis set was used in *Dmol 3*. The orbital cutoff quality is to be set as "Fine" in this calculation. The charge density difference was calculated as regards COF coordinated with metal ion (Zn, Cu, Ni, Co, Fe and Mn). In order to simplify the calculation difficulty and shorten the calculation time, the monolayer models were used for all calculations.

The free energy of the adsorbed state (ΔG_{H*}) was calculated using the Gaussian 09W program. During geometry and frequency optimization, all atoms were allowed to move freely. Based on the Density Functional Theory (DFT), the quantum cluster calculations were carried out using the B3LYP/6-31G (d, p) basis set. The SMD solvation model was used to consider the solvent (water) effect.

The adsorption energy was calculated according to the following equation:

$$\Delta E_{ad} = E_{Metals-Salen\,COF_{EDA}/H_{ads}} - E_{Metals-Salen\,COF_{EDA}} - \frac{1}{2}E_{H_2} \qquad \text{Equation (2)}$$

Where: $E_{\text{Metal-Salen COF EDA/Hads}}$ is the total energy of Metal-Salen COF_{EDA} with absorption of H; $E_{\text{Metal-Salen COF EDA}}$ is the energy of the Metal-Salen COF_{EDA} surface; E_{H2} is the energy of hydrogen in the gas phase.

Gibbs free energy was calculated by considering zero-point energy (ZPE) and entropy corrections for the hydrogen evolution reaction as per the following equation:[4]

$\Delta G_H = \Delta E_{ad} + \Delta E_{ZPE} - T\Delta S$ Equation (3)

Where ΔE_{ad} is obtained from Equation (2). In the adsorbed state, hydrogen shows negligible entropy change due to vibrational force, so the Gibbs free energy is calculated by considering the following corrections:[4]

$\Delta G_H = \Delta E_{ad} + 0.24$ eV Equation (4)

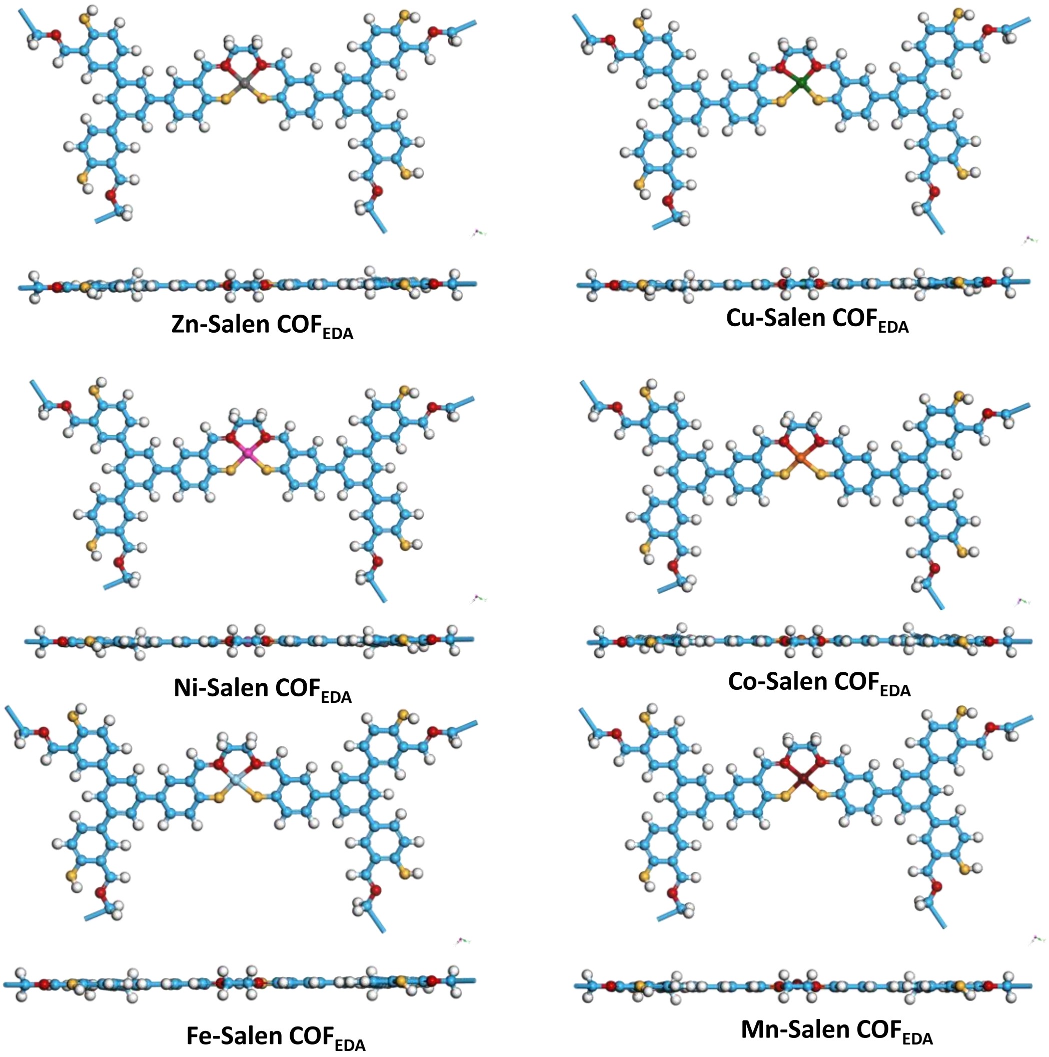

Figure S17. Proposed models for Metal-Salen COF$_{EDA}$ for calculating DOS and charge density difference (H atom: white, C atom: cyan, N atom: read, O atom: yellow, Zn atom: grey, Cu atom: green, Ni atom: pink, Co atom: orange, Fe atom: light blue, Mn atom: wine red).

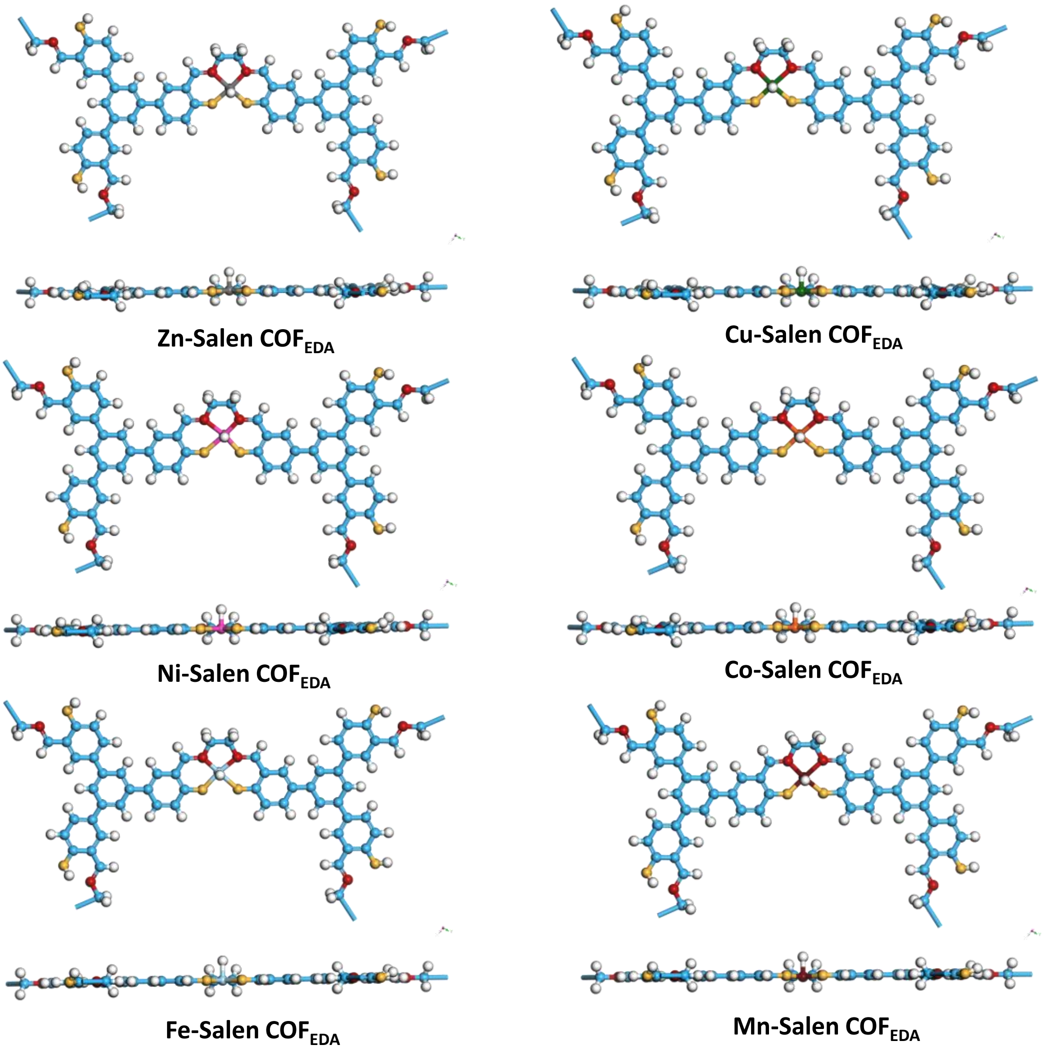

Figure S18. Models for Metal-Salen COF_{EDA} after adsorbing the H atom (H atom: white, C atom: cyan, N atom: read, O atom: yellow, Zn atom: grey, Cu atom: green, Ni atom: pink, Co atom: orange, Fe atom: light blue, Mn atom: wine red).

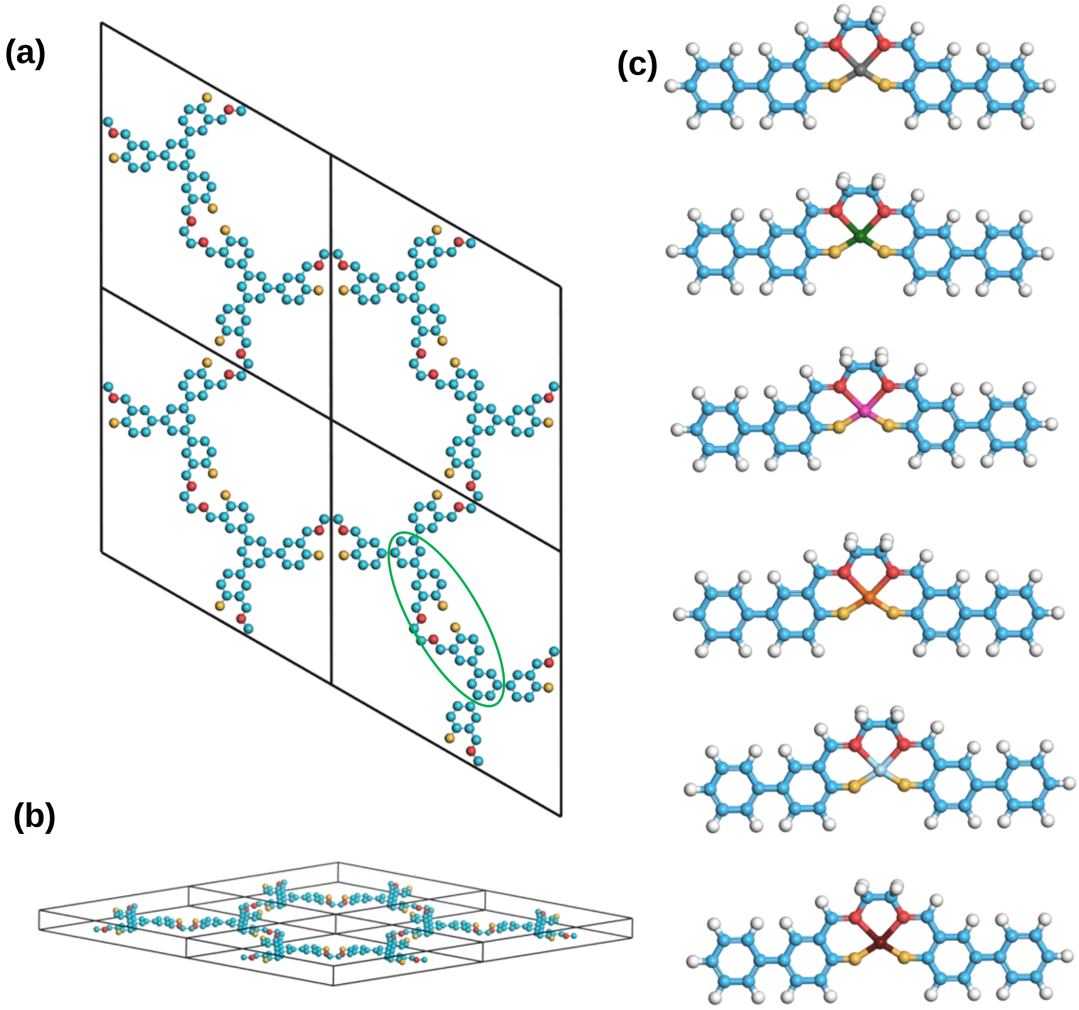

Figure S19 (a) Optimized geometric structure of the 2 × 2 × 1 supercell of Salen COF_{EDA}. (b) Three-dimensional view of Salen COF_{EDA}. (c) Geometric structure of simplified model catalysts (H atom: white, C atom: cyan, N atom: read, O atom: yellow, Zn atom: grey, Cu atom: green, Ni atom: pink, Co atom: orange, Fe atom: light blue, Mn atom: wine red).

The unite cell of hexagonal crystal system is indicated by the box (black). The metal-free model structure of cluster calculation is indicated by the oval box (green). Each independent model is provided to clear where the cut was made and what the capping group is.

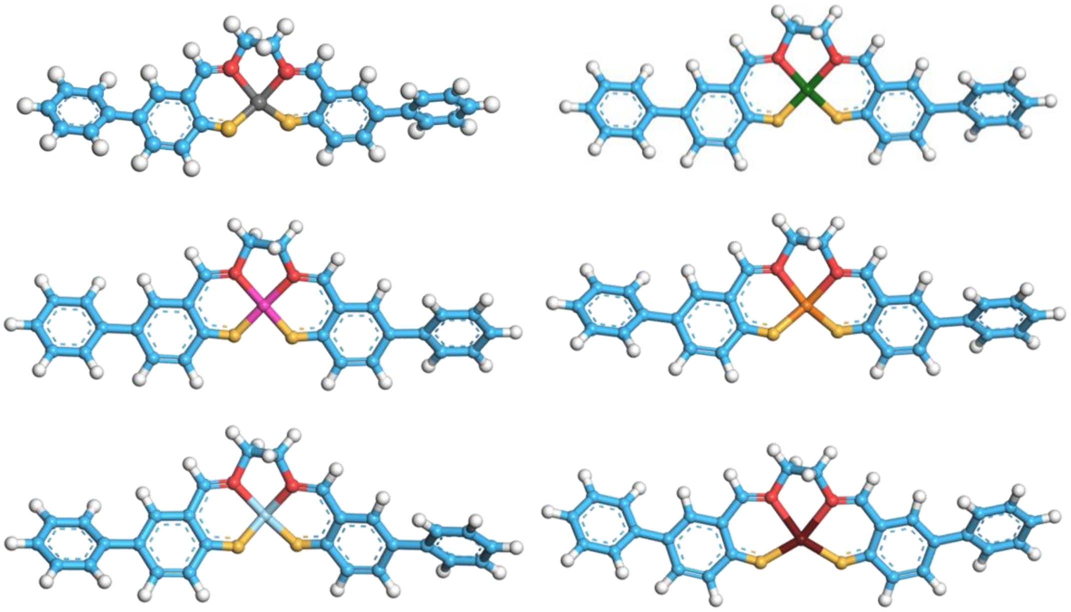

Figure S20. Geometric structure after cluster calculations (H atom: white, C atom: cyan, N atom: read, O atom: yellow, Zn atom: grey, Cu atom: green, Ni atom: pink, Co atom: orange, Fe atom: light blue, Mn atom: wine red).

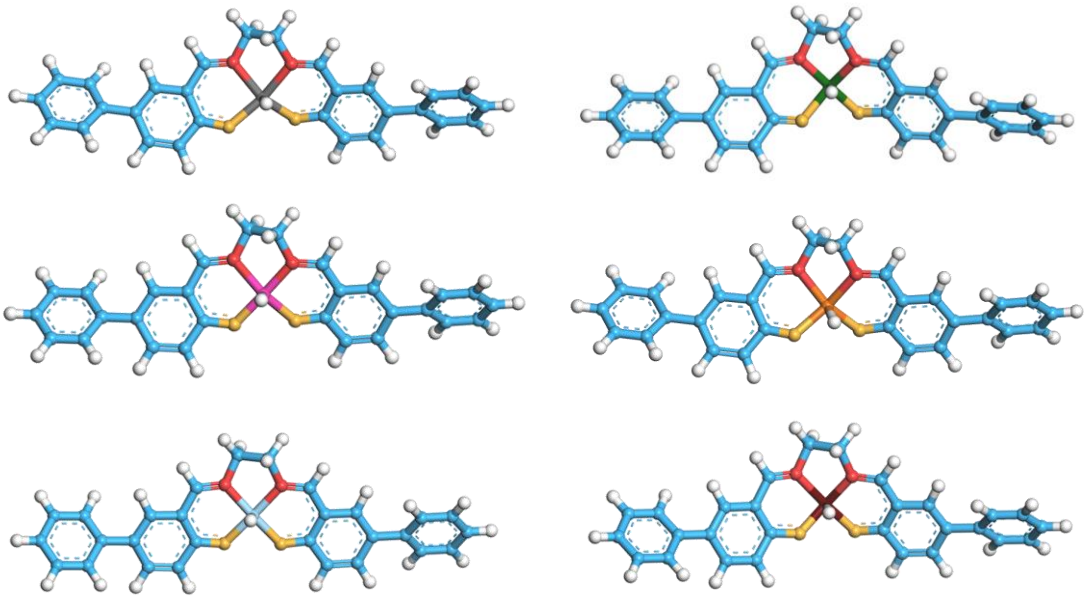

Figure S21. Adsorbed H geometric structure after cluster calculations (H atom: white, C atom: cyan, N atom: read, O atom: yellow, Zn atom: grey, Cu atom: green, Ni atom: pink, Co atom: orange, Fe atom: light blue, Mn atom: wine red).

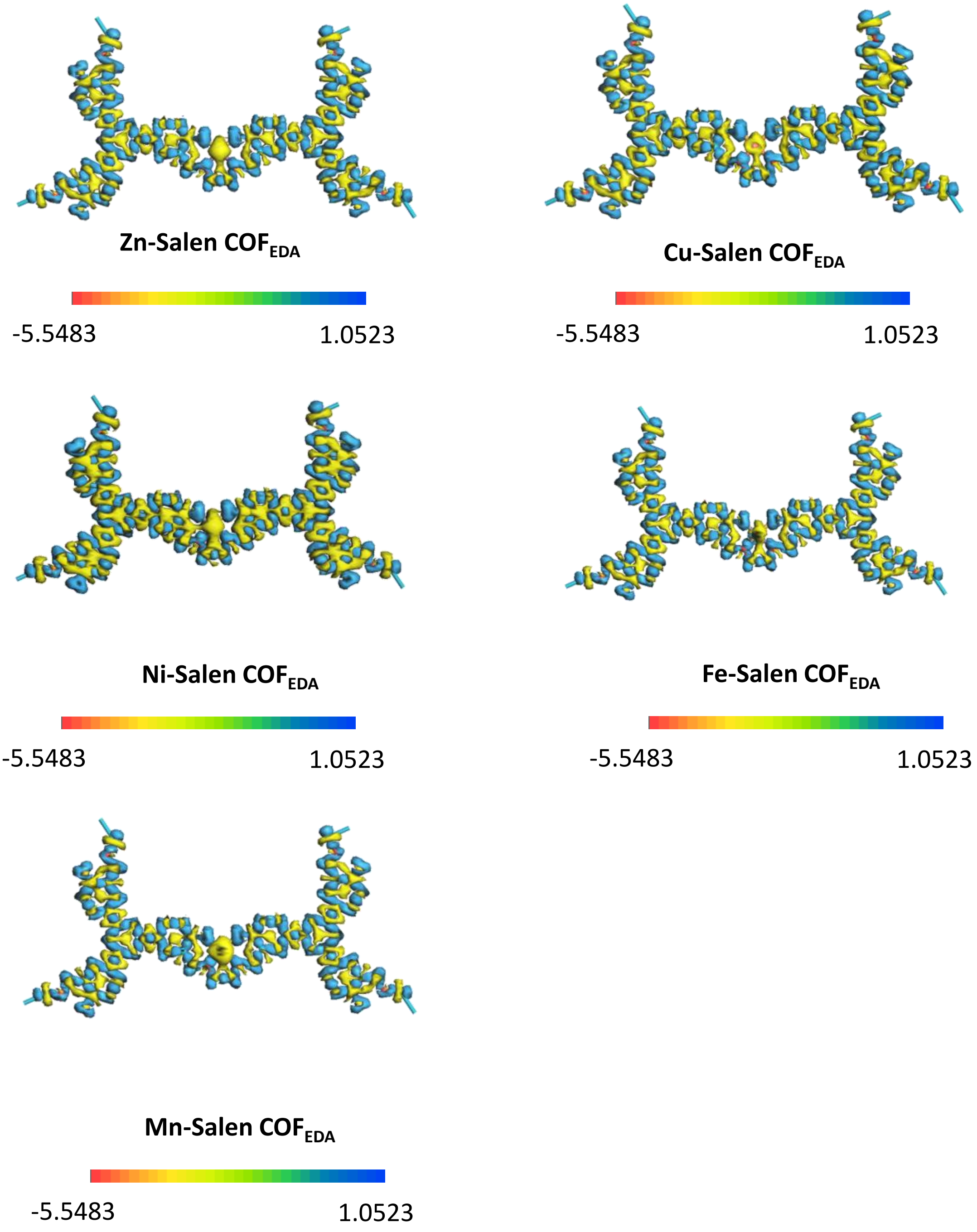

Figure S22. Calculated charge density difference for the Zn-Salen COF$_{EDA}$, Cu-Salen COF$_{EDA}$, Ni-Salen COF$_{EDA}$, Fe-Salen COF$_{EDA}$ and Mn-Salen COF$_{EDA}$.

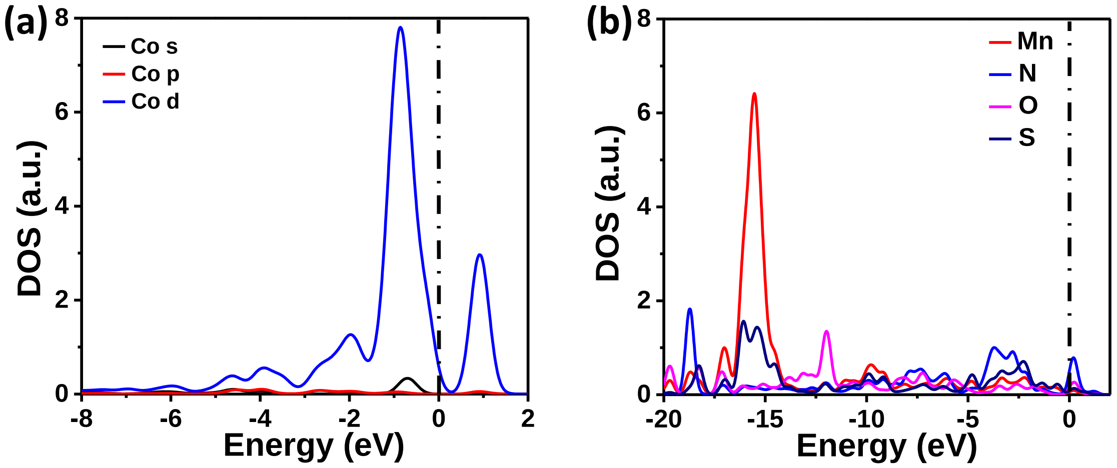

Figure S23. Calculated PDOS of: (a) Co atom in Co-Salen COF_{EDA} (the black dashed line denotes the position of the Fermi level); (b) PEDOT@Mn-Salen COF_{EDA}.

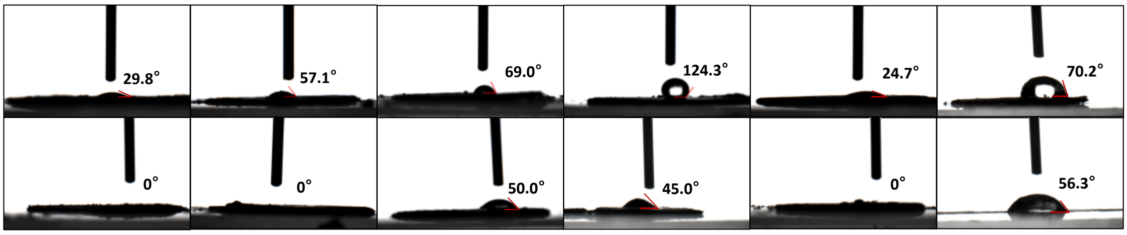

Figure S24. Contact angle of Metal-Salen COF_{EDA} and PEDOT@Mn-Salen COF_{EDA}.

Table S1. Fractional atomic coordinates for the unit cell of Salen COF_{EDA}.

Atom	X	Y	Z
C1	0.36893	0.6849	0.50009
C2	0.33548	0.70419	0.50009
C3	0.38507	0.61255	0.50009
C4	0.36573	0.56038	0.50009
C5	0.39942	0.54134	0.50009
C6	0.45373	0.57519	0.50009
C7	0.47316	0.62718	0.50009
C8	0.43918	0.64575	0.50009
O9	0.48824	0.55834	0.50009
C10	0.39837	0.38035	0.50009
N11	0.45157	0.40217	0.50009
C12	0.3759	0.4866	0.50009
C13	0.57816	0.015	0.50009
N14	0.55357	0.04038	0.50009
C15	0.28162	0.67013	0.50009
C16	0.2607	0.61755	0.50009
C17	0.35618	0.75939	0.50009
C18	0.41009	0.79474	0.50009
C19	0.42975	0.84695	0.50009
C20	0.39466	0.8642	0.50009
C21	0.341	0.82918	0.50009
C22	0.3219	0.77723	0.50009
O23	0.41197	0.91501	0.50009
C24	0.48674	0.88178	0.50009
C25	0.29484	0.59895	0.50009
C26	0.34913	0.63235	0.50009
C27	0.2034	0.58257	0.50009
C28	0.17049	0.60262	0.50009
C29	0.11634	0.57035	0.50009
C30	0.09423	0.51653	0.50009
C31	0.12675	0.49629	0.50009
C32	0.18098	0.52899	0.50009
O33	0.04131	0.48354	0.50009
C34	0.0856	0.59488	0.50009
C35	0.95749	0.57176	0.50009
N36	0.92533	0.51833	0.50009
C37	0.6772	0.37057	0.50009
C38	0.69633	0.33815	0.50009
C39	0.60395	0.38477	0.50009
C40	0.55016	0.36593	0.50009

C41	0.53117	0.39863	0.50009
C42	0.56686	0.45146	0.50009
C43	0.62042	0.47043	0.50009
C44	0.63885	0.43741	0.50009
O45	0.55021	0.48511	0.50009
C46	0.38162	0.41471	0.50009
N47	0.40497	0.46536	0.50009
C48	0.47422	0.37604	0.50009
C49	0.00818	0.59139	0.50009
N50	0.03431	0.56613	0.50009
C51	0.66098	0.2845	0.50009
C52	0.60733	0.26287	0.50009
C53	0.75265	0.36004	0.50009
C54	0.78822	0.41452	0.50009
C55	0.84128	0.43554	0.50009
C56	0.85921	0.40121	0.50009
C57	0.82401	0.34694	0.50009
C58	0.77118	0.32652	0.50009
O59	0.91082	0.41982	0.50009
C60	0.87597	0.49308	0.50009
C61	0.58899	0.29601	0.50009
C62	0.62361	0.34994	0.50009
C63	0.5711	0.20613	0.50009
C64	0.59058	0.17416	0.50009
C65	0.55742	0.12065	0.50009
C66	0.50321	0.09822	0.50009
C67	0.48348	0.12986	0.50009
C68	0.51712	0.18346	0.50009
O69	0.46917	0.04584	0.50009
C70	0.5817	0.09106	0.50009
C71	0.56256	0.96349	0.50009
N72	0.50941	0.93083	0.50009

Section S4. Reference List

[1] X. Han, Q. Xia, J. Huang, Y. Liu, C. Tan, Y. Cui, *J. Am. Chem. Soc.* **2017**, *139*, 8693.

[2] H.-B. Liu, M. Wang, Y. Wang, L. Wang, L.-C. Sun, *Synthetic Commun.* **2010,** *40*, 1074.

[3] Y. Wu, D. Yan, Z. Zhang, M. M. Matsushita, K. Awaga, *ACS Appl. Mater. Inter.* **2019**, *11*, 7661.

[4] M. D. Hossain, Z. Liu, M. Zhuang, X. Yan, G.-L. Xu, C. A. Gadre, A. Tyagi, I. H. Abidi, C.-J. Sun, H. Wong, A. Guda, Y. Hao, X. Pan, K. Amine, Z. Luo, *Adv. Energy Mater.* **2019,** *9*, 1803689.
